# Dibasic Derivatives of Phenylcarbamic Acid as Prospective Antibacterial Agents Interacting with Cytoplasmic Membrane

**DOI:** 10.3390/antibiotics9020064

**Published:** 2020-02-06

**Authors:** Šárka Pospíšilová, Ivan Malík, Kristyna Bezouskova, Tereza Kauerova, Peter Kollar, Jozef Csöllei, Michal Oravec, Alois Cizek, Josef Jampilek

**Affiliations:** 1 Regional Centre of Advanced Technologies and Materials, Faculty of Science, Palacky University, Slechtitelu 27, 783 71 Olomouc, Czech Republic; sharka.pospisilova@gmail.com; 2 Department of Pharmaceutical Chemistry, Faculty of Pharmacy, Comenius University in Bratislava, Odbojarov 10, 832 32 Bratislava, Slovakia; 3 Department of Infectious Diseases and Microbiology, Faculty of Veterinary Medicine, University of Veterinary and Pharmaceutical Sciences, Palackeho 1946/1, 612 42 Brno, Czech Republic; bezouskova.kristyna@gmail.com (K.B.); cizeka@vfu.cz (A.C.); 4 Department of Human Pharmacology and Toxicology, Faculty of Pharmacy, University of Veterinary and Pharmaceutical Sciences, Palackeho 1, 61242 Brno, Czech Republic; tereza.kauerova@gmail.com (T.K.); kollarp@vfu.cz (P.K.); 5 Department of Chemical Drugs, Faculty of Pharmacy, University of Veterinary and Pharmaceutical Sciences in Brno, Palackeho 1946/1, 612 42 Brno, Czech Republic; csolleij@vfu.cz; 6 Global Change Research Institute CAS, Belidla 986/4a, 603 00 Brno, Czech Republic; oravec.m@czechglobe.cz; 7 Institute of Neuroimmunology, Slovak Academy of Sciences, Dubravska cesta 9, 845 10 Bratislava, Slovakia

**Keywords:** carbamate, antibacterial, synergy, antibiofilm activity, structure–activity relationships

## Abstract

1-[2-[({[2-/3-(Alkoxy)phenyl]amino}carbonyl)oxy]-3-(dipropylammonio)propyl]pyrrolidinium/azepan- ium oxalates or dichlorides (alkoxy = butoxy to heptyloxy) were recently described as very promising antimycobacterial agents. These compounds were tested in vitro against *Staphylococcus aureus* ATCC 29213, *Enterococcus faecalis* ATCC 29212 (reference and control strains), three methicillin-resistant isolates of *S. aureus*, and three isolates of vancomycin-resistant *E. faecalis*. 1-[3-(Dipropylammonio)-2-({[3-(pentyloxy-/hexyloxy-/heptyloxy)phenyl]carbamoyl}oxy)propyl]pyrrolidinium dichlorides showed high activity against staphylococci and enterococci comparable with or higher than that of used controls (clinically used antibiotics and antiseptics). The screening of the cytotoxicity of the compounds as well as the used controls was performed using human monocytic leukemia cells. IC_50_ values of the most effective compounds ranged from ca. 3.5 to 6.3 µM, thus, it can be stated that the antimicrobial effect is closely connected with their cytotoxicity. The antibacterial activity is based on the surface activity of the compounds that are influenced by the length of their alkoxy side chain, the size of the azacyclic system, and hydro-lipophilic properties, as proven by in vitro experiments and chemometric principal component analyses. Synergistic studies showed the increased activity of oxacillin, gentamicin, and vancomycin, which could be explained by the direct activity of the compounds against the bacterial cell wall. All these compounds demonstrate excellent antibiofilm activity, when they inhibit and disrupt the biofilm of *S. aureus* in concentrations close to minimum inhibitory concentrations against planktonic cells. Expected interactions of the compounds with the cytoplasmic membrane are proven by in vitro crystal violet uptake assays.

## 1. Introduction

Since the 1970s, there is an increasing tendency to study the antibacterial activity of drugs belonging to different pharmaceutical groups not recognized as antimicrobials. These compounds are called non-antibiotics [1,2]. Their common feature is the ability to modify cell permeability; thus, they are also called membrane stabilizers [3]. There is overwhelming evidence for the antibacterial activity of phenothiazines and their derivatives as well as for their synergistic effect with antibiotics [1,4,5].

The antibacterial effect of local anesthetics has been known for a long time [6,7,8,9,10,11,12]. Mullin et al. [13] performed an in vitro study to test the antibacterial activity of commercially available topical anesthetics. Kesici et al. [14] compared the antibacterial effect of bupivacaine and prilocaine. Efficiency against *Staphylococcus aureus* and *Escherichia coli* was notably significantly higher when prilocaine was used. Pina-Vaz et al. [15] reported the antifungal concentration-dependent activity of benzydamine, lidocaine, and bupivacaine against twenty *Candida* strains. Srisatjaluk et al. [16] studied the effect of lidocaine against oral flora and discovered that 10% spray possessed bactericidal activity against *E. coli*, *Streptococcus salivarus*, and *Streptococcus sanguinis*, which increased with exposure time. Several studies reporting the combined effect of local anesthetics with preservatives, antiseptics, opioids, or intravenous anesthetics are available [17,18,19], but the number of synergistic studies with antibiotics is limited [20]. The mechanisms of the antibacterial activity of local anesthetics are not completely known, but based on the ability of surfactants to interact with the cells by several different mechanisms, such as insertion into lipid bilayers, modification of membrane permeabilization via channel formation, or modification of membrane solubilization [21], it can include disruption of bacterial membrane, inhibition of cell wall synthesis, alteration in DNA synthesis, inhibition of membrane bound enzymatic activities, and many others [20].

Approximately 65% of bacterial infections are associated with biofilm formation [22]. An important part of biofilm infections, apart from such things as wound infections or internal organ infections resulting from pathogen migration from another infected organ, such as endocarditis, is related to indwelling medicinal devices, such as central venous catheters, mechanical heart valves, peritoneal dialysis catheters, and prosthetic or urinary catheters [23,24,25,26]. Catheter-associated urinary tract infections (CAUTI) are the most common cause of secondary blood infections [27]. Catheter-related bloodstream infections are mostly caused by coagulase-negative staphylococci, *S. aureus*, enteric Gram-negative bacilli, and *Candida* spp. [28]. Coating catheter surfaces with different organic and inorganic materials is a way to prevent catheter-associated infections. A nitrofurazone-impregnated catheter is the only commercially available catheter covered with organic material [27]. A randomized clinical trial performed by Menezes et al. did not show any benefits of using nitrofurazone-coated urinary catheters compared to non-impregnated silicon catheters [29]. An appropriate lubricant should be used during catheter insertion to minimize urethral trauma and infection. Instilagell^®^ contains chlorhexidine and 4-hydroxybenzoic acid synergistically reducing bacterial biofilm and lidocaine, which provides comfort to a patient following the procedure [30,31]. Lai et al. compared the results from 57 randomized controlled trials studying the effect of catheter impregnation on central-venous catheter-related infections in adults [32]. The study consisted of 11 types of impregnations and 16,784 catheters. The antimicrobial impregnations of central venous catheters did not reduce clinically diagnosed sepsis and all-cause mortality but reduced the rate of catheter-related bloodstream infections. The benefits of central venous catheter impregnation by antimicrobial agents for the reduction of catheter-related bloodstream infections are also discussed in other papers (e.g., [33,34,35,36]). Similarly, the importance of catheter coating by antimicrobials for the prevention of catheter-associated urinary tract infections is described in other papers as well (e.g., [37,38,39,40]).

Staphylococci are one of the most frequent causes of nosocomial infections and infections related to biofilm formation. Compared to *Staphylococcus epidermidis*, infections caused by *S. aureus* biofilm are more difficult to treat [41,42]. Despite the commonly accepted idea that biofilms are 100-fold more resistant than planktonic cells [43,44,45], there are also studies showing the opposite [46,47]. The main reasons for the higher resistance of microbial pathogens to antibiotics are as follows [48]: (i) lower antibiotic penetration due to the extra-polymer matrix [45,49,50]; (ii) producing enzymes that modify the activity of antibiotics [51]; (iii) persistent cells [44]; (iv) nutritional limitations [52]; and stress responses [53].

This study is a follow-up paper to a recently published article [54] describing a synthesis of dibasic derivatives of phenylcarbamic acid, their physicochemical properties, and antimycobacterial activity. These compounds were originally designed as local anesthetics with favorable solubility. The most active derivatives were a hundred times more active than standard procaine [55]. The general chemical structure of these compounds is comparable to the structures of the above-mentioned non-antibiotics, all of which contain lipophilic groups, polar moieties, hydrocarbon connecting chains, and nitrogen(s)-containing salt-forming fragments [54], as seen in Table 1. Since the anticipated antimycobacterial activities of some of these derivatives are proven [54], it was decided to extend the knowledge about their ability to fight microorganisms in respect to Gram-positive pathogens. Thus, the current study is aimed at the description of the complex activity of 1-[2-[({[2-/3-(alkoxy)phenyl]-amino}carbonyl)oxy]-3(dipropylammonio)propyl]pyrrolidinium/azepan- ium oxalates/dichlorides against *S. aureus* and *Enterococcus faecalis* including some multidrug-resistant isolates. Minimum inhibitory concentration (MIC) against planktonic cells as well as the bactericidal and synergistic effect of selected compounds were assessed. In addition, minimum biofilm inhibitory concentration and the effect of the compounds against preformed biofilm were investigated.

## 2. Results and Discussion

### 2.1. Chemistry

The synthesis and physicochemical descriptors of the presently investigated 1-[2-[({[2-/3-(alkoxy)- phenyl]amino}carbonyl)oxy]-3-(dipropylammonio)propyl]pyrrolidinium/azepanium oxalates or dichlorides (**1a**–**1p**; alkoxy = butoxy to heptyloxy) were published previously [54,55]. The chemical structures of the compounds together with their selected physicochemical characteristics (i.e., lipophilic (log *k*_w_), surface (γ), and electronic (log ε_2 (Ch-T)_) properties), are listed in Table 1.

### 2.2. Antibacterial Activity

All the compounds were evaluated in vitro against *S. aureus* ATCC 29213 and *E. faecalis* ATCC 29212 as reference and quality control strains and subsequently against three clinical isolates of methicillin-resistant *S. aureus* (MRSA) and three isolates of vancomycin-resistant *E. faecalis* (VRE) [56]. In addition, the series of compounds were tested against yeast strain *Candida albicans* CCM 8261 (Table 2). 1-[3-(Dipropylammonio)-2-({[3-(heptyloxy)phenyl]carbamoyl}oxy)propyl]pyrrolidinium dichloride (**1h**) and 1-[3-(dipropylammonio)-2-({[3-(hexyloxy)phenyl]carbamoyl}oxy)propyl]pyrrolidinium dichloride (**1g**) were the only compounds strongly effective against all the tested pathogens. Good antibacterial activity was observed for 1-[3-(dipropylammonio)-2-({[3-(pentyloxy)phenyl]carbamoyl}- oxy)propyl]pyrrolidinium dichloride (**1f**). Azepanium derivatives **1m**–**1p** showed medium activity against enterococci (Table 2). The evaluation of minimum fungistatic activity against *C. albicans* was added to compare the activity against bacterial and yeast cells. Only compounds **1h** and **1g** showed antifungal activity (Table 2). In addition, the compounds were tested against Gram-negative pathogen *E. coli* ATCC 25922 and showed no activity (MIC >256 µg/mL, data not shown).

The antimicrobial effect of the dibasic derivatives of phenylcarbamic acid depended on the length of the alkoxy tail (i.e., the effect is dependent on surface activity as described recently [54,57,58,59,60] and discussed below). This observation is also consistent with the results of the antibacterial activity of local anesthetics published by Pere et al. [61]. In addition, it seems that the activity was influenced by a heterocyclic salt-forming moiety. All highly effective compounds **1f**–**1h** contained a pyrrolidinium ring, which was more advantageous than the presence of azepan-1-yl (i.e., balanced hydro-lipophilic properties play a role, as discussed below). It should be noted that more lipophilic azepanium ring-containing derivatives demonstrated comparable or higher antimycobacterial activities than pyrrolidinium moiety-containing molecules [54]. In addition, 3-alkoxy positional isomers were more efficient against tested microorganisms than the 2-alkoxy ones.

### 2.3. In Vitro Antiproliferative Assay

The preliminary in vitro screening of the antiproliferative effect of the investigated compounds was performed using a Water Soluble Tetrazolium salts-1 (WST-1) assay kit [62] and a human monocytic leukemia THP-1 cell line by means of the method described recently [63]. The principle of the WST-1 assay kit is the inhibition of mitochondrial dehydrogenases by antiproliferative compounds. The activity of this enzyme directly correlates with the number of metabolically active cells in the culture. The antiproliferative effect was evaluated as the IC_50_ value (the concentration of the compound causing 50% inhibition of cell proliferation). IC_50_ values of the most effective compounds, **1h**, **1g**, **1f**, **1p** and **1o**, ranged from ca. 3.5 to 6.3 µM (Table 2). The IC_50_ of camptothecin was ca. 0.20 µM, and the IC_50_ values of other antiseptics used as control agents were ca. 1.29 and 1.73 µM (Table 2). Thus, it can be stated that the antimicrobial effect is closely connected with their cytotoxicity. On the other hand, ciprofloxacin (CPX) showed the most significant antiproliferative effect on THP-1 cells (IC_50_ = 0.71 ± 0.09 µM), as described recently [64]. Finally, we conclude that all the dibasic derivatives of phenylcarbamic acid as well as other antiseptics are considered cytotoxic agents, since according to literature (e.g., [65]), a compound is considered cytotoxic if it shows a toxic effect on cells up to 10 μM.

### 2.4. Structure–Activity Relationships

Similarities, differences, or grouping patterns between the physicochemical descriptors of compounds **1a**–**1p** estimated previously [49], in vitro antimicrobial efficiency, and in vitro antiproliferative (cytotoxic) effect against the human monocytic leukemia THP-1 cell line were analyzed using unscaled principal component analysis (PCA). A set of new orthogonal variables, called principal components (PCs) and a pattern of similarity of observations were the results of the analysis [66].

Chemometrically processed physicochemical parameters were represented by surface tension γ (relative surface activity; in N/m units), logarithms of molar absorption coefficients log ε_2 (Ch-T)_ of their methanolic solutions, which were observed in the UV–Vis region of an electromagnetic spectrum, as well as extrapolated lipophilicity indices log *k*_w_ obtained by isocratic reversed-phase (RP)-HPLC (Table 1). The antibacterial activity of the compounds was expressed as log(1/MIC (M)), and their antiproliferative (cytotoxic) effect was described as IC_50_ values (in μM units). Biological results related to *Candida albicans* CCM 8261 were not included in the analysis because only two compounds (**1g** and **1h**) showed notable efficiency against given yeast (Table 2). The analysis was carried out by the XLSTAT software, ver. 2016.02.28451.

In order to put the analyzed physicochemical and biological (antimicrobial and antiproliferative) indices on the same scale, several data pretreatment methods were investigated [67,68,69]. The number of PCs was determined using the visual evaluation of a scree plot as a relationship between calculated eigenvalues (λ_e_) and the number of PCs. The λ_e_ descriptor measured the amount of variations retained by each PC [67]. The proper selection of relevant PCs was based on the Kaiser–Guttman rule [70]. The first three interpreted PCs of the analysis accounted for 89.41% of the total variance in the data as follows: PC 1 (57.52%, λ_e_ = 6.33), PC 2 (22.19%, λ_e_ = 2.44), and PC 3 (9.70%, λ_e_ = 1.07). The PCs did not prove the existence of ‘real’ parameters; they only indicated that the existence of these descriptors was mathematically possible.

The relationship between PC 1 and PC 2 resulted in the division of all examined compounds **1a**–**1p** into typical subgroups. Those PCs accounted for the majority of the data variability (79.71% in total). Differences in compounds´ physicochemical and in vitro biological properties were reflected in PC 1 and PC 2 values. Clear distinction between biologically active and ineffective derivatives could be made according to PC 1.

**Figure 1:**
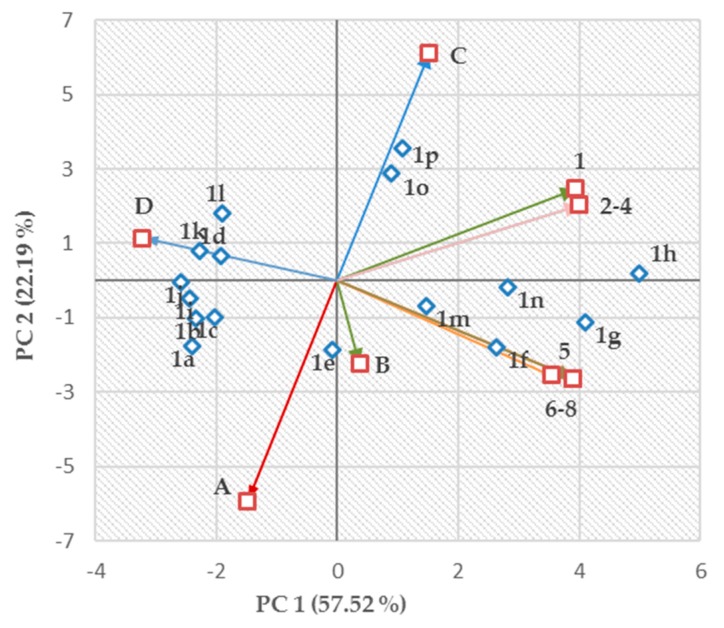
Two-dimensional (2D) score plot (mapping) showing (i) both principal component 1 (PC 1) and 2 (PC 2) scores of compounds **1a**–**1p**; (ii) loadings of variables (i.e., variously colored vectors), the assignment (numbering) of which is as follows: A (vector assigned to the variable γ), B (log ε_2 (Ch-T)_), C (log *k*_w_), D (*IC*_50_), 1 (vector built on log (1/MIC (M)) values, which were connected with the in vitro testing of compounds **1a**–**1p** against *E. faecalis*), 2 (VRE 368), 3 (VRE 725B), 4 (VRE 342B), 5 (SA 29213), 6 (MRSA SA 3202), 7 (MRSA SA 630), and 8 (MRSA 63718).

One subgroup, which was defined by PC 1 > 0.00, included 3-alkoxy substituted molecules **1f**–**1h** and **1m**–**1p** with notable activity against at least three bacterial strains. On the other hand, those compounds showed significant antiproliferative (cytotoxic) effect with IC_50_ < 6.50 μM (Figure 1).

A closer look into the formed subgroups led to more precise conclusions. The most lipophilic derivatives, **1o** (log *k*_w_ = 5.6569) and **1p** (log *k*_w_ = 6.1749), which had (i) the highest ability to fight *E. faecalis*, VRE 342B and VRE 725B, (ii) insignificant efficiency against *S. aureus* (SA) ATCC 29213, MRSA 63718, MRSA SA 630, and MRSA SA 3202, and (iii) IC_50_ < 6.50 μM against the human monocytic leukemia THP-1 cell line showed PC 1 > 0.00 together with PC 2 > 2.89. These molecules were located on the upper right side of a two-dimensional (2D) score plot (Figure 1). In addition, both compounds, together with highly antiproliferative acting agent **1h** (IC_50_ = 3.67 ± 0.01 μM, PC 1 = 5.00, PC 2 = 0.20), showed the highest ability to decrease the surface tension of water varying from 0.05692 N/m (**1p**) to 0.05925 N/m (**1o**; Table 1). The derivatives, which were effective against all the tested bacterial strains (**1f**–**1h**) or against almost all the bacteria (**1m**, **1n**), having significant antiproliferative (cytotoxic) effect (IC_50_ < 5.00 μM), were located on the bottom right side (**1f**, **1g**) or in positions closer to the PC 1 axis (**1m**, **1n**, **1h**) of the score plot with PC 2 from 0.02 (**1h**) to −1.79 (**1f**; Figure 1).

Conversely, the 2-alkoxy substituted molecules with the lowest in vitro activity against all the tested strains and IC_50_ > 10.00 μM were included in the second subgroup located in the left upper and bottom quadrants of the score plot (**1a**–**1d**, **1i**–**l**). All those substances were defined by PC 1 < 0.00. In fact, antimicrobially efficient 3-butoxy derivative **1e** with significant antiproliferative (cytotoxic) activity (IC_50_ = 4.20 ± 0.79 μM) was found at the ´edge´ within the score plot, as proven by PC 1 = −0.09 (Figure 1).

The ability of derivatives **1a**, **1c**, **1b**, **1i**, **1j**, **1d**, **1k**, and **1l** to decrease the surface tension of water and increase their lipophilicity was connected with increasing PC 2 (PC 1 < 0.00).

In addition, the compounds with the most favorable toxicological properties located in the left upper quadrant (**1k**, **1l**) showed PC 2 > 0.80 and PC 1 < −1.90 (Figure 1). The most in vitro active compounds, however, with considerable antiproliferative (cytotoxic) ability, showed PC 2 in the interval from −1.13 (**g**) to 3.55 (**p**) as well as PC 1 > 0.00 (Figure 1).

**Figure 2:**
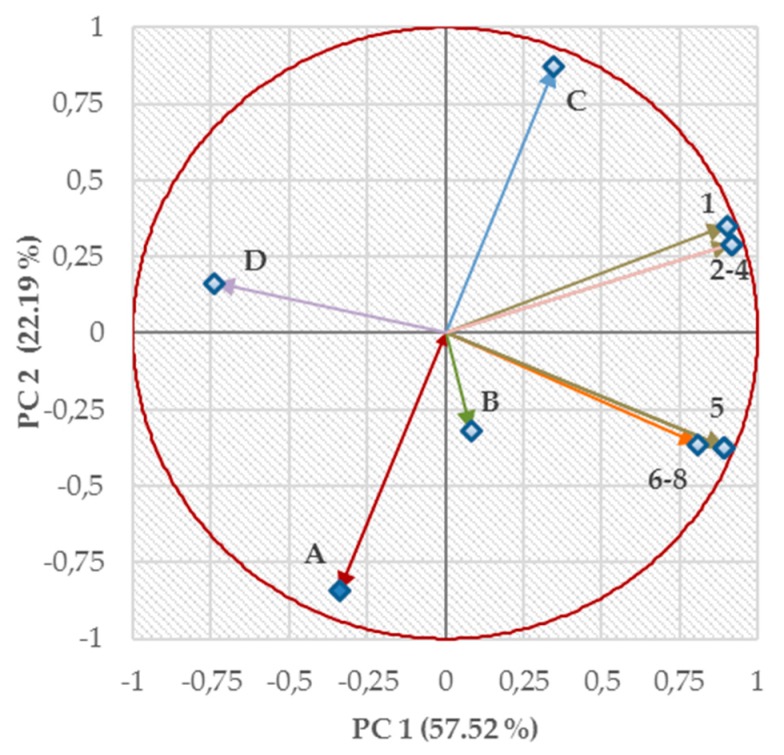
2D mapping of loadings of variables (variously colored vectors) indicating their (i) positions towards a circle of correlation and (ii) relationships with both principal component 1 (PC 1) and 2 (PC 2). The assignment (numbering) of the vectors is as follows: A (vector assigned to the variable γ), B (log ε_2 (Ch-T)_), C (log *k*_w_), D (IC_50_), 1 (vector built on log (1/MIC (M)) values, which are connected with the in vitro testing of compounds **1a**–**1p** against *E. faecalis*), 2 (VRE 368), 3 (VRE 725B), 4 (VRE 342B), 5 (SA 29213), 6 (MRSA SA 3202), 7 (MRSA SA 630), and 8 (MRSA 63718).

When the data set was properly pretreated, the quality of 2D representation of a variable was visualized by the distance between the projected variable onto a plane and the circle of correlation. The loadings of particular variables (i.e., physicochemical or biological descriptors), defined the size of the contribution of each original variable to particular PCs [66]. The loadings were indicated as variously colored vectors and assigned (or numbered) according to their position in the score plot (Figure 1). In more detail, the letter A was assigned to the variable γ and digit 1 was related to the vector built on the log(1/MIC (M)) values, which were connected with the in vitro testing of compounds **1a**–**1p** against *E. faecalis*. Analogously, the assignment or numbering of other vectors was based on their physicochemical or biological ‘nature’ as follows: B (log ε_2 (Ch-T)_), C (log *k*_w_), D (IC_50_) 2 (VRE 368), 3 (VRE 725B), 4 (VRE 342B), 5 (SA 29213), 6 (MRSA SA 3202), 7 (MRSA SA 630), and 8 (MRSA 63718). These colored vectors (loadings) were also assigned (or numbered) following their position in the circle of radius 1 in an absolute value (Figure 2). Regarding this comprehensive circle of correlation, visual assessment indicated the smallest angles between vectors 5–8. Relatively sharp angles were also observed between vectors 1–4, and their mutual relationships were described by values of Pearson’s correlation coefficient (*r*) [71]. It was assumed that molecules **1a**–**1p** would show similar mechanisms of action against (i) *E. faecalis* and VRE strains (*r* = 0.981) or (ii) SA 29213 and MRSA strains (*r* = 0.891). The visualizations on axes PC 1 and PC 2 (Figure 2) revealed a ‘certain’ connection between lipophilicity (log *k*_w_; vector C) and efficiency against *E. faecalis* (1), VRE 368 (2), VRE 725B (3), or VRE 342B (4). However, lipophilicity was not considered a decisive factor influencing the activity of screened molecules, as proven by calculated *r* values for those Gram-positive bacteria (vectors) as follows: 1 (*r* = 0.566), 2 (*r* = 0.499), 3 (*r* = 0.499), and 4 (*r* = 0.499). Similar trends were found between surface properties (γ; vector A) and efficiency against *E. faecalis* (1), VRE 368 (2), VRE 725B (3), or VRE 342B (4) and proved by *r* = −0.456 (VRE strains) and −0.532 (*E. faecalis*).

It seems that the ‘almost orthogonal’ arrangement of the vector characterizing surface properties (A) in relation to activities against *Staphylococcus* spp. (5–8) indicated a quite questionable correlation (Figure 2). The corresponding *r* values were too low: −0.032 (vectors 6–8) and −0.107 (5). Similar behaviors were observed when exploring the relationships between lipophilicity (C) and activities against the SA strains (5–8). The relationships were characterized by *r* = 0.032 (5) and 0.052 (6–8). In addition, negatively correlated variables (vectors) were observed and positioned in the opposed quadrants of the 2D loading plot (Figure 2). These correlations were aimed at γ (A) versus log *k*_w_ (C; *r* = −0.938), A versus 1 (*r* = −0.532), and A versus 2, 3, or 4 (*r* = −0.456 in all cases). A vector that was built on electronic properties (log ε_2 (Ch-T)_; B) was not defined quite sufficiently in both PC 1 and PC 2. The contribution of the variable to PC 1 and PC 2 was only 0.11% and 4.13%, respectively. In this case, PC 3 together with PC 4 were considered the most suitable components, and the log ε_2 (Ch-T)_ variable contributed with 51.13% to PC 3 and 42.72% to PC 4.

The IC_50_ values (D) were most notably (negatively) correlated with activity against VRE strains (*r* = −0.738). In other words, the ability of tested compounds to fight given bacteria might be connected with their antiproliferative (cytotoxic) potential. It was also observed that the potency against methicillin-susceptible (*r* = −0.498) or resistant (*r* = −0.585) *S. aureus* strains were only partially connected with the antiproliferative (cytotoxic) activity. Antiproliferative (cytotoxic) effect might also be regarded as independent of surface (*r* = 0.003), electronic (*r* = −0.065), and lipophilic properties (*r* = 0.062).

### 2.5. Advanced Antimicrobial Evaluation

#### 2.5.1. Synergistic Effect

The most active agents, **1g** and **1h**, were studied in combination with clinically used antibacterial drugs (Table 3). Representatives from various classes were chosen in order to study the potential difference in synergistic activity according to the diverse mechanisms of action and resistance of these drugs. The method of minimal fractional inhibitory concentration (FIC) index (FICI) in a microtitration plate was used. FICI ≤ 0.5 means synergy; 0.5 < FICI < 1 means additivity; 1 ≤ FICI < 4 means indifference; and FICI ≥ 4 means antagonism [72,73].

Synergistic activity was observed for the combinations of compound **1g** with oxacillin (OXA) against MRSA 63718 and compound **1h** with vancomycin (VAN) against VRE 342B. The combinations of compound **1g** with VAN against VRE 342B and compound **1h** with OXA against MRSA 63718 possessed additivity with the FIC index close to the limit of synergy (0.562 and 0.625, respectively). Both OXA and VAN are antibiotics interacting with cytoplasmic membrane and cell wall, but in a different step of peptidoglycan synthesis [74,75]. The mechanism of resistance of bacteria to VAN and OXA differs as well; VAN-resistant enterococci replace the terminal d-Ala of peptidoglycan precursors with d-lactate, which decreases the affinity of VAN 1000-fold [76]. OXA resistance is provided by the expression of different penicillin-binding proteins PBP2a [77]. The availability of these compounds to increase the activities of both above-mentioned antibiotics is explained by their direct interaction with the cytoplasmic membrane and cell wall.

The used VRE strains were isolated from American crows by Oravcova et al. [56], and their genetic profiles were characterized. All the VRE strains were carrying genes *vanA*, *tetM*, and *ermB*. Strains VRE 368 and VRE 725B carried gene *aac* [56], which provided acquired aminoglycoside resistance. In contrast to the intrinsic resistance, the acquired resistance cannot be overcome using combinations with cell wall-active drugs, such as VAN or penicillin antibiotics [78]. The difference in the activity of the combination with gentamicin (GEN) against VRE strains could be caused by the different types of aminoglycoside resistance of these strains. As agents capable of interacting with the cell wall, the compounds can increase the penetration of GEN to the bacterial cells and increase its activity. The combination of **1g** and **1h** with VAN against VRE 725B had an indifferent effect (data not shown). No combination with GEN against VRE 725B was tested due to the high MIC of GEN (>2000 µg/mL).

#### 2.5.2. Time-Kill Studies

Time-kill studies are used to evaluate the dynamics of antibacterial activity. As mentioned above, a pre-test to determine minimum bactericidal concentration (MBC) by sub-cultivation of aliquots on agar was made (see Section 3.4). All the active compounds possessed bactericidal activity, which means that their MBCs were ≤ 4× MICs [79] (data not shown). Compounds **1g** and **1h** were chosen to study the dynamics of antibacterial activity because they had the highest in vitro potency (Table 4, Table 5). The agents were tested in concentrations equal to 1× MIC, 2× MIC, and 4× MIC. Time-kill studies were made with methicillin-susceptible *S. aureus* ATCC 29213 and methicillin-resistant MRSA 63718, vancomycin-susceptible *E. faecalis* ATCC 29212, and all three VRE isolates. Vancomycin was used as a control (Table 6).

Compound **1g** (see data in Table 4) showed bactericidal effects only against *E. faecalis* in concentration 2× MIC 4 and 6 h after the start of the incubation and in concentration 4× MIC at 4, 6, and 8 h. Killing ≥90 % of colonies was observed for VRE 725B, VRE 368, and MRSA 63718 at least at one time and in one concentration. Results were statistically analyzed using two-way ANOVA followed by Tukey’s test (see Section 3.8). The interaction between concentration and time was significant for the activity against MRSA 63718 and VRE 368. Antibacterial effect against *S. aureus* and VRE 342B was based on time and concentration, but no significant relationship between these parameters was observed. For VRE 725B, only concentration was important to achieve an antibacterial effect. In general, the statistically significant antibacterial effect of the compounds was observed 4 h from the start of the incubations, which supported the theory based on the interaction(s) of the compounds with cytoplasmic membrane.

No bactericidal effect was observed in the case of compound **1h** (for data see Table 5). If a statistically significant decrease in colony-forming unit (CFU)/mL was achieved compared to the growth control at time 0, this would be detected immediately after 4 h from the start of incubation. Interaction between time and concentration was significantly important in all cases excluding VRE 342B and VRE 368. These observations could support the expected mechanism of action of compound **1h** as well.

Although compounds **1g** and **1h** possessed bactericidal activity in the test of subcultivation of aliquot on agar plates, the results of time-kill studies did not prove this. The discrepancy could be caused by the use of different methods: microtiter (MBC assay) and macrobroth dilutions (time-kill studies) and also by different growth phases of the organisms used.

#### 2.5.3. Crystal Violet Uptake

**Figure 3:**
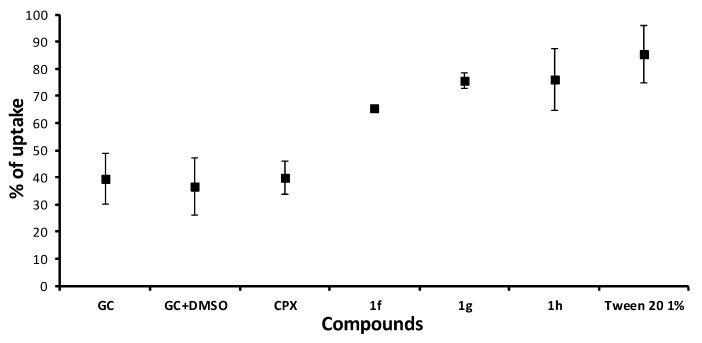
Crystal violet uptake of **1f**, **1g**, and **1h** (64 µg/mL) treated with *S. aureus* ATCC 29213. Means ± SD for three replicates are illustrated. GC = growth control; GC + DMSO = growth control with DMSO equal to the concentration of DMSO in tested tubes; CPX = ciprofloxacin.

The alternation in membrane permeability was detected by crystal violet assay [80]. Bacterial suspension treated by compounds **1f**, **1g**, and **1h** (all 64 µg/mL) for 1 h was prepared. The uptake of crystal violet was expressed as a percentage compared to the original crystal violet solution. Results are illustrated in Figure 3.

CPX, as an agent non-interacting with the cytoplasmic membrane, was used as a negative control, while 1% solution of surface active Tween 20 was used as a positive control. The effect of CPX on violet uptake was comparable to the uptake of non-treated cells *(p* < 0.05). All of the tested dibasic phenylcarbamates possessed strong effect for increasing crystal violet uptake. Results were statistically analyzed using two-way ANOVA followed by Tukey’s test. No statistically significant difference (*p* < 0.05) was found between either compounds **1f**, **1g**, and **1h** or between the tested compounds and 1% Tween 20, which means that those dibasic esters of 3-alkoxyphenylcarbamic acid significantly influenced membrane permeability.

#### 2.5.4. Antibiofilm Activity

**Figure 4:**
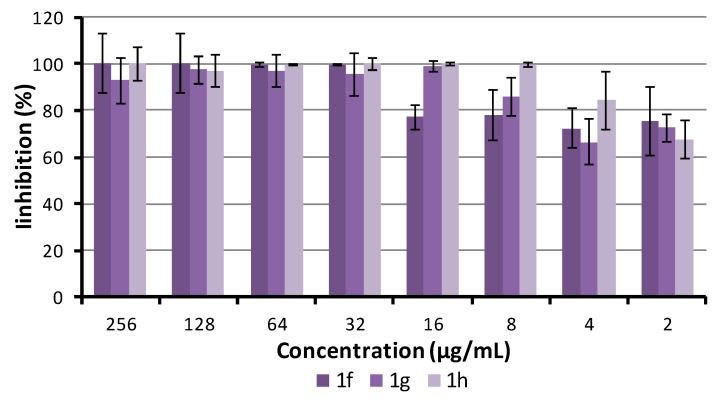
Inhibition activity of compounds **1f**, **1g**, and **1h** on *S. aureus* ATCC 29213 biofilm.

**Figure 5:**
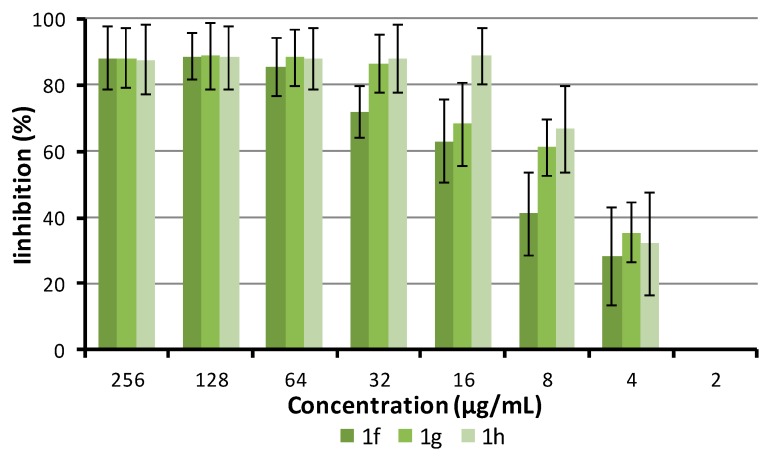
Disrupting effect of compounds **1f**, **1g**, and **1h** on *S. aureus* ATCC 29213 biofilm formed.

The ability of compounds **1f**, **1g**, and **1h** to inhibit biofilm growth and destroy the matured biofilm of *S. aureus* ATCC 29213 was investigated. The inhibitory activity was studied by the method with crystal violet [81]. The activity to disrupt matured biofilm was evaluated as a decrease of metabolic activity using the MTT assay. The minimum biofilm inhibitory control, which inhibited ≥80% of biofilm formation compared to the growth control, was equal to minimum inhibitory concentration against planktonic cells in the case of compounds **1g** and **1h** and two-fold higher in the case of **1f** (see Figure 4). All the tested compounds showed 80% degradation of preformed biofilm in concentrations that were only two-fold (for compound **1h**) or four-fold higher (**1g**, **1f**) than MIC (see Figure 5). The activity of the compounds increased with the increasing surface activity and the length of the alkyl chain.

Clinically used local anesthetics (procaine, trimecaine, and tetracaine), antiseptics (carbethopendecinium bromide, cetalkonium chloride), and anionic surfactant sodium dodecyl sulfate were used as controls of biofilm inhibition and disruption (Table 7). The biofilm inhibitory concentration of cation-active disinfection carbethopendecinium bromide was comparable to its MIC, but the eradication concentration was 128-fold higher. Cetalkonium chloride did not show any activity on preformed biofilm. Thus, it is concluded that the eradication effect of the studied compounds on premature staphylococcal biofilm does not only depend on the surface activity.

## 3. Materials and Methods

### 3.1. Chemistry

All the investigated compounds **1a**–**1p** were synthesized and characterized recently [54,55].

### 3.2. Minimum Inhibitory Concentration Determination

The modified broth microdilution method [72,82] was used for in vitro evaluation of minimum inhibitory concentration. A small portion of bacterial colony cultivated overnight onto nutrient agar (Oxoid, Basingstoke, UK) with 5% bovine blood was suspended in sterile phosphate-buffered saline to get cell density 0.5 McFarland. This inoculum was diluted to reach the final concentration of bacterial cells 5 × 10^5^ CFU/mL in the wells. The compounds were dissolved in DMSO and diluted in the broth to get final concentrations 256–2 µg/mL in the wells. For plates with staphylococci, cation adjusted Mueller–Hinton (CaMH) broth (Oxoid) was used; enterococci were cultivated in brain heart infusion (BHI, Oxoid). Ampicillin (AMP) and ciprofloxacin (CPX), purchased from Sigma (Poole, UK), were used as reference drugs. A drug-free control and a sterility control were included. Inoculated plates were incubated in aerobic atmosphere at 37 °C overnight. The minimum inhibitory concentration (MIC) was evaluated as the lowest concentration of tested compounds, which inhibited 100% of visual growth of the bacteria. The test was made in triplicate; the results are shown in Table 2. After evaluation of MICs, 10 µL of aliquots from the wells were put onto Mueller–Hinton agar. The agar plates were cultivated at 37 °C for 24 h. The minimum bactericidal concentration (MBC) was defined as the lowest concentration of the compound that killed 99.9% of the bacteria compared to the starting inoculum.

Fungistatic activity was tested similarly in RPMI (Roswell Park Memorial Institute) 1640 medium [72] (Sigma, Pool, UK). Tested compounds dissolved in DMSO were diluted in RPMI-1640 to final concentrations 128–4 µg/mL. A growth control and sterility control were included. The colonies of *C. albicans* grown overnight on Sabourad dextrose agar were suspended in sterile phosphate-buffered saline (PBS) to get cell density 0.5 McFarland. The inoculum was diluted to get a final concentration of 10^3^ CFU/mL in the wells. Plates were inoculated and cultivated at 37 °C for 48 h. The minimum fungistatic concentration was visually evaluated as the lowest concentration of a tested compound, which inhibited 100% of yeast growth. 5-Flucytosin (Sigma, UK) was used as a positive control. The test was made in triplicate; the results are shown in Table 2.

### 3.3. Synergy Effect with Clinically Used Drugs

For synergy effect study, a method of fractional inhibitory concentration was used. A tested compound (A) and a conventional used antibiotic (B) (oxacillin, gentamicin, ciprofloxacin, and vancomycin, purchased from Sigma) were diluted in the microtitration plate in CaMH broth or BHI (Oxoid) to get an original combination of concentrations in every well. Raw H was used for the evaluation of MIC(A); column 12 was used for evaluation of MIC(B). The plate was inoculated by the bacterial suspension to reach final concentration of 5 × 10^5^ CFU/mL in the wells. The fractional inhibitory concentration (FIC) index was calculated using the concentrations in the first nonturbid (clear) well found in each row and column along the turbidity/nonturbidity interface. To interpret the combined effect, the lowest FICI was used [72]. A ΣFIC ≤ 0.5 means synergy; 0.5 < ΣFIC < 1 means additivity; 1 ≤ ΣFIC < 4 means indifference; and ΣFIC ≥ 4 means antagonism [73]. The tests were made in duplicate, and the results were averaged. The results are summarized in Table 3.

### 3.4. Time-Kill Study

The method of time-kill curves [72] was used to study the bactericidal effect of selected compounds. For this experiment, only compounds **1g** and **1h** with high antibacterial activity were used. The experiment was performed with *S. aureus* ATCC 29213, MRSA 63718, *E. faecalis* ATCC 29212, and all VRE isolates. The compounds were diluted in CaMH broth (for staphylococci) or BHI broth (for enterococci) to reach concentrations equal to 1 × MIC, 2 × MIC, and 4 × MIC. The tubes were inoculated by bacterial inoculum in the exponential phase of growth to get a final concentration of 7.5 × 10^6^ CFU/mL. The tubes were incubated statically at 37 °C. Immediately after inoculation and after 4, 6, 8, and 24 h, 100 µL of the sample was serially diluted (1:10) in PBS. Then, 2 × 20 µL from each dilution were put onto a Mueller–Hinton agar plate and cultivated at 37 °C for 24 h. After incubation, CFUs of dilutions containing 5–50 colonies were counted. Results were expressed as a decrease of log_10_(CFU) in each time compared to the starting inoculum. Bactericidal effect is defined as a −3log decrease of CFU/mL compared to the growth control in time 0. The test was made in duplicate on two separate occasions, and the results were averaged. The results of the decrease of log_10_(CFU) are shown in Table 4, Table 5 and Table 6. The growth curves with error bars are shown in Figures S1 and S2 in Supplementary Materials.

### 3.5. Crystal Violet Uptake

The method of crystal violet uptake [80] was used to study membrane alteration. Bacterial suspension was cultivated to log phase in CaMH and then harvested at 4500 rpm for 5 min. The cells were washed twice and resuspended in PBS containing 64 µg/mL of the tested compounds. Additionally, 1% Tween 20 and ciprofloxacin (64 μg/mL) were used as controls. A growth control without antibiotics and a control containing the same amount of DMSO as treated tubes were included. The tubes were cultivated at 37 °C for 1 h. After that, the tubes were centrifuged at 4500 rpm for 15 min and washed twice in PBS. The cells were resuspended in PBS containing crystal violet (10 µg/mL). After 15 min incubation at 37 °C and centrifugation (15 min, 4500 rpm), the absorbance of supernatant at 595 nm was measured. The experiment was repeated three times, and the results were averaged. The percentage of crystal violet uptake was counted according to the equation:

(1) $$\%\;of\;uptake\;=\;\frac{OD_{595}\;of\;sample}{OD_{595}\;of\;crystal\;violet\;solution}\;\times \;100$$

### 3.6. Biofilm Inhibition Assay

Compounds **1f**, **1g**, and **1h**, showing the highest antistaphylococcal activity, were studied as inhibitors of biofilm formations. These derivatives were diluted in a 96-well plate in tryptic soya broth (TSB) containing 2% glucose, and their final concentrations were 256–2 µg/mL. The plates were inoculated by inoculum of *S. aureus* ATCC 29213 grown in TSB + 2% glucose to exponential phase. Before the inoculation of the plate, the original inoculum was diluted to 1 McFarland and then 1:100 in fresh TSB + 2% glucose, to reach the final concentrations in the wells of 1 × 10^5^. As the compounds were dissolved in DMSO (up to 2.5%), the growth control included 2.5% of DMSO for verification that the applied DMSO concentration did not inhibit growing of the bacterial biofilm. The plates were incubated at 37 °C for 48 h. After incubation, the contents of the wells were removed, and the plates were washed three times by sterile PBS. After drying, 125 μL of 0.5% crystal violet was added to each well and the plates were stained at room temperature for 20 min. Then the dye was removed, and the plates were washed three times by sterile PBS. The colored biofilm was detached from the wells using 33% solution of acetic acid. The absorbance at 595 nm was measured. As a blank, a non-inoculated plate treated in the same way was used. The ability to inhibit biofilm formation was evaluated as a percentage inhibition of growth compared to the growth control according to the equation:

(2) $$\%\;of\;inhibition\;=\;100\;-\;\frac{OD_{595S}}{OD_{595C}}\;\times \;100$$

where *OD_595S_* is the absorbance of sample at 595 nm and *OD_595C_* is the absorbance of growth control at 595 nm. The minimum biofilm inhibitory concentration was the lowest concentration of the compounds, which inhibited the growth of 80% bacteria compared to the growth control. The experiment was made in duplicate and repeated at least three times.

### 3.7. Biofilm Susceptibility to Compounds

Biofilms were grown as described above but without the presence of the compounds. After 48 h of incubation at 37 °C, the contents of the wells were removed, and the plates were washed three times by sterile PBS. After washing, 100 µL of tested compounds in CaMH were added to the wells. Concentrations of the compounds ranged from 256 to 2 µg/mL. A growth control containing 2.5% of DMSO in CaMH was included. The plates were incubated at 37 °C for 24 h, then the solutions of compounds were removed, and the plates were gently washed three times by sterile PBS. Bacterial viability was analyzed using 100 µL of 0.05 mg/mL MTT (3-(4,5-dimethylthiazol-2-yl)- 2,5-diphenyltetrazolium bromide) in PBS in each well. The plates with MTT were incubated at 37 °C for 1–2 h in darkness, until the blue formazan crystals appeared. MTT solution was removed and the plates were washed once by PBS. Crystals of formazan were dissolved using 17% sodium dodecysulphate in 40% dimethylformamid. The absorbance at 570 nm was measured and the percentage of eradication was counted according to the equation

(3) $$\%\;of\;eradication\;=\;100\;-\;\frac{OD_{570S}}{OD_{570C}}\;\times \;100$$

where *OD_570S_* is the absorbance of the sample at 570 nm and *OD_570C_* is the absorbance of the growth control at 570 nm. The minimum biofilm eradication concentration was the lowest concentration of the compound, which reduced the metabolic activity of the biofilm by 80% compared to the growth control. The experiment was made in duplicate and repeated at least three times.

### 3.8. Calculations and Statistical Analyses

Chemometric principal component analysis (PCA), a powerful multivariate statistical technique, was used to investigate a set of values, in which observations were described by several inter-correlated quantitative dependent variables. The PCA tool was used to explore relationships between physicochemical descriptors (γ, log ε_2 (Ch-T)_, log *k*_w_), in vitro activity (in log(1/MIC (M) units) against Gram-positive bacterial strains, and antiproliferative (cytotoxic) properties (IC_50_ values in μM units) of compounds **1a**–**1p**. The physicochemical or biological data connected with standard drugs (i.e., AMP, CPX, VAN, and camptothecin), were not included in the calculations.

PCA is a mathematical algorithm that reduces the dimensionality of the data, while retaining most of the variation in a data set. It accomplishes this reduction by identifying directions, called principal components (PCs), along which the variation in the data is maximum. By using a few components, each sample is represented by relatively few numbers instead of thousands of variables. Samples are then plotted, making it possible to visually assess similarities and differences between samples and determine whether samples can be grouped [83]. In order to put all the analyzed physicochemical and biological indices on the same scale, several data pretreatment methods were investigated (i.e., standardized [67] and centered [68] transformation procedures as well as Pareto scaling [69] were applied). The resulting values of all relevant PCA-based descriptors, but not the values of transformed variables, were the same if considering all the given rescaling techniques. The proper definitions and meaning of the used terms are provided below. A scree plot (not provided) was used to plot eigenvalues according to their size and visualize if there was a point in this graph such that the slope of the graph went from ‘steep’ to ‘flat’ and to keep only the components, which were before the elbow. The eigenvalue (λ_e_) associated to a component is equal to the sum of the squared factor scores for this component [66]. The Kaiser–Guttman rule is considered the most common stopping rule in PCA [70], which is aimed at an average value of *λ*_e_ > 1.0. Circle of correlation is defined as the set of points, the sum of squared coordinates which is equal to a constant. Consequently, when the data are perfectly represented by only two components, the sum of the squared loadings is equal to one, and therefore, in this case, the loadings are positioned on the circle [66]. Pearson’s correlation coefficient (*r*) is a measure of the strength of a linear relationship between two variables, which indicates a positive or negative correlation as a measure of reliability [71]. The PCA was performed by the XLSTAT software, ver. 2016.02.28451 (Addinsoft, New York, NY, USA), a cloud-based statistical application for statistics and data analyses, which was used as an add-on to Microsoft Excel 2013 (Microsoft Corp., Redmont, WA, USA).

The effect of concentration and time on the antibacterial activity of the compounds studied by time-kill curves was analyzed by the two-way ANOVA (*p* < 0.05) with the Tukey´s test for multiple comparison. The analysis was performed with OriginPro 2018 SR1 Build 195 software (OriginLab Corporation, Northampton, MA, USA).

### 3.9. In Vitro Antiproliferative Assay

Human monocytic leukemia THP-1 cells were used for in vitro antiproliferative assay. Cells were obtained from the European Collection of Cell Cultures (ECACC, Salisbury, UK) and routinely cultured in RPMI (Roswell Park Memorial Institute) 1640 medium supplemented with 10% fetal bovine serum, 2% l-glutamine, 1% penicillin, and streptomycin at 37 °C with 5% CO_2_. Cells were passaged at approximately one-week intervals. The antiproliferative activity of the compounds was determined using a Water Soluble Tetrazolium Salts-1 (WST-1, 2-(4-iodophenyl)-3-(4-nitrophenyl)-5-(2,4-disulfophenyl)-2*H*-tetrazolium) assay kit (Roche Diagnostics, Mannheim, Germany) according to the manufacturer’s instructions [62]. The tested compounds were dissolved in DMSO and added in various compound concentrations to the cell suspension in the culture RPMI 1640 medium. The maximum concentration of DMSO in the assays never exceeded 0.1%. Subsequently, the cells were incubated at 37 °C with 5% CO_2_ for 24 h. For WST-1 assays, cells were seeded into 96-well plates (5 × 10^4^ cells/well in 100 μL culture medium) in triplicate in serum-free RPMI 1640 medium, and measurements were taken 24 h after the treatment with the compounds. The median inhibition concentration values, IC_50_, were deduced through the production of a dose-response curve. All data were evaluated using GraphPad Prism 5.00 software (GraphPad Software, San Diego, CA, USA). The results are shown in Table 2.

## 4. Conclusions

Sixteen dibasic esters of 2-/3-alkoxyphenylcarbamic acid (**1a**–**1p**; alkoxy = butoxy to heptyloxy) originally prepared as effective local anesthetics were tested in vitro against Gram-positive pathogens (i.e., three methicillin-resistant isolates of *S. aureus*, three isolates of vancomycin-resistant *E. faecalis*, *S. aureus* ATCC 29213, and *E. faecalis* ATCC 29212 being a reference and control strain). In addition, minimum fungistatic activity against *C. albicans* CCM 8261 was investigated in vitro. 1-[3-(Dipropylammonio)-2-({[3-(heptyloxy)phenyl]carbamoyl}oxy)propyl]pyrrolidinium dichloride (**1h**) and 1-[3-(dipropylammonio)-2-({[3-(hexyloxy)phenyl]carbamoyl}oxy)propyl]pyrrolidinium dichloride (**1g**) were the most effective against bacterial as well as fungal pathogens. Good antibacterial activity was also observed for 1-[3-(dipropylammonio)-2-({[3-(pentyloxy)phenyl]carbamoyl}oxy)propyl]pyrrolidin- ium dichloride (**1f**). Regarding chemometric principal component analysis (PCA) results, compounds **1a**–**1p** showed similar mechanisms of action against (i) *E. faecalis* and VRE strains (VRE 342B, VRE 725B) or (ii) SA 29213 and MRSA strains. A ‘certain’ linear relationship was observed between lipophilicity (log *k*_w_) and efficiency against *E. faecalis* (1), VRE 368 (2), VRE 725B (3), or VRE 342B (4). However, lipophilicity would not be a decisive factor influencing the activity of the screened molecules, which was proven by calculated *r* values for the vectors describing compounds´ activity against those Gram-positive bacteria as follows: 1 (*r* = 0.566), 2 (*r* = 0.499), 3 (*r* = 0.499), and 4 (*r* = 0.499). Similar trends were found between surface properties (γ) and efficiency against *Enterococcus* spp. and described by *r* = −0.456 (VRE strains) and −0.532 (*E. faecalis*). The ability of molecules **1a**–**1p** to fight given bacteria might be related to their antiproliferative (cytotoxic) potential, which was independent of surface (*r* = 0.003), electronic (*r* = −0.065), and lipophilic properties (*r* = 0.062). On the other hand, the potency against methicillin-susceptible or resistant *S. aureus* strains was only partially connected with antiproliferative (cytotoxic) features, described by the values of *r* = −0.498 and −0.585. Surface properties would also contribute to activity against given bacteria. Synergistic in vitro studies showed an increase in activity of oxacillin and vancomycin, which could be explained by the direct activity of screened dibasic 2-/3-alkoxyphenylcarbamates against the bacterial cell wall. Compound **1g** decreased the MIC value of gentamicin in the case of vancomycin-resistant *E. faecalis* with intrinsic aminoglycoside resistance, which also supported the theory of the interaction with a cell wall. All these compounds had excellent antibiofilm activity, when inhibiting and disrupting the biofilm of *S. aureus* in the concentrations close to MICs against planktonic cells. The expected interaction of the investigated compounds with a cytoplasmic membrane was proven by the uptake of crystal violet to the bacterial cells after treatment by given compounds. On the other hand, this mechanism of action is also responsible for cytotoxicity, which is a limiting factor for the eventual use of the compounds in vivo. As the compounds possessed strong activity to prevent biofilm formation, the potential application could be the development of antibacterial-coated catheters. The synergistic potential with clinically used antibiotics could decrease the needed concentration to reach an antibacterial effect, which would reduce their cytotoxic potential. The strong anesthetic activity of the compound is also beneficial. In summary, several studied derivatives showed high antibacterial activity against Gram-positive bacteria including multidrug-resistant isolates and the ability to fight biofilm colonization. Overall, dibasic esters of substituted phenylcarbamic acid might be regarded as promising for future research in the field of local antibacterial drugs.

## Figures and Tables

**Table 1:** Chemical structure of investigated compounds **1a**–**1p** determined lipophilicity (log *k*_w_) estimated by isocratic reversed-phase (RP)-HPLC, surface tension γ (relative surface activity (N/m)), and logarithms of molar absorption coefficients (log ε_2 (Ch-T)_) of methanolic solutions (*c* = 8.0 × 10^−5^ M) of individual compounds (taken from Malik et al. [54]).

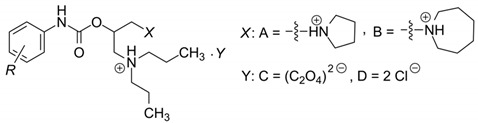						
Comp.	R	X	Y	log k_w_	γ (N/m)	log ε_2 (Ch-T)_
**1a**	2-OC_4_H_9_	A	C	3.7688	0.06464	4.19
**1b**	2-OC_5_H_11_	A	C	4.0454	0.06366	4.05
**1c**	2-OC_6_H_13_	A	C	4.6049	0.06222	4.52
**1d**	2-OC_7_H_15_	A	C	4.9487	0.05985	4.08
**1e**	3-OC_4_H_9_	A	D	4.0258	0.06316	4.24
**1f**	3-OC_5_H_11_	A	D	4.6722	0.06285	4.27
**1g**	3-OC_6_H_13_	A	D	4.9446	0.06105	4.13
**1h**	3-OC_7_H_15_	A	D	5.5384	0.05786	4.27
**1i**	2-OC_4_H_9_	B	C	4.4679	0.06302	4.08
**1j**	2-OC_5_H_11_	B	C	4.8466	0.06206	4.22
**1k**	2-OC_6_H_13_	B	C	5.2359	0.06065	4.10
**1l**	2-OC_7_H_15_	B	C	5.8966	0.05853	4.14
**1m**	3-OC_4_H_9_	B	D	4.7099	0.06298	4.09
**1n**	3-OC_5_H_11_	B	D	5.2087	0.06154	4.18
**1o**	3-OC_6_H_13_	B	D	5.6569	0.05925	4.01
**1p**	3-OC_7_H_15_	B	D	6.1749	0.05692	4.20

**Table 2:** In vitro minimum inhibitory concentrations (MICs, µg/mL (µM)) of compounds **1a**–**1p** against tested microbial strains compared to control agents and in vitro antiproliferative (Tox) data (IC_50_ (μM)) compared to controls.

Comp.	MICs (µg/mL (µM))									Tox IC_50_ (μM)
	SA	MRSA1	MRSA2	MRSA3	EF	VRE1	VRE2	VRE3	CA	
**1a**	>256 (>427)	>256 (>427)	>256 (>427)	>256 (>427)	>256 (>427)	>256 (>427)	>256 (>427)	>256 (>427)	>128 (>213)	10.80 ± 1.11
**1b**	>256 (>417)	>256 (>417)	>256 (>417)	>256 (>417)	>256 (>417)	>256 (>417)	>256 (>417)	>256 (>417)	>128 (>208)	12.53 ± 1.22
**1c**	>256 (>407)	>256 (>407)	>256 (>407)	>256 (>407)	>256 (>407)	>256 (>407)	>256 (>407)	>256 (>407)	>128 (>203)	15.42 ± 0.22
**1d**	>256 (>398)	>256 (>398)	>256 (>398)	>256 (>398)	>256 (>398)	>256 (>398)	>256 (>398)	>256 (>398)	>128 (199)	16.32 ± 0.70
**1e**	64 (129)	64 (129)	64 (129)	64 (129)	128 (259)	32 (65.1)	64 (129)	32 (65.1)	>128 (>259)	4.20 ± 0.79
**1f**	**16** **(31.6)**	**16** **(31.6)**	**32** **(63.2)**	**16** **(31.6)**	**32** **(63.2)**	**16** **(31.6)**	**16** **(31.6)**	**16** **(31.6)**	**128** **(252)**	4.19 ± 0.54
**1g**	**8** **(15.4)**	**8** **(15.4)**	**16** **(30.8)**	**8** **(15.4)**	**16** **(30.8)**	**8** **(15.4)**	**8** **(15.4)**	**8** **(15.4)**	**16** **(30.8)**	3.50 ± 0.31
**1h**	**8** **(15.0)**	**8** **(15.0)**	**8** **(15.0)**	**8** **(15.0)**	**8** **(15.0)**	**4** **(7.51)**	**4** **(7.51)**	**4** **(7.51)**	**8** **(15.0)**	3.67 ± 0.01
**1i**	>256 (>407)	>256 (>407)	>256 (>407)	>256 (>407)	>256 (>407)	>256 (>407)	>256 (>407)	>256 (>407)	>128 (>203)	23.06 ± 0.95
**1j**	>256 (>398)	>256 (>398)	>256 (>398)	>256 (>398)	>256 (>398)	>256 (>398)	>256 (>398)	>256 (>398)	>128 (>199)	26.45 ± 1.91
**1k**	>256 (>390)	>256 (>390)	>256 (>390)	>256 (>390)	>256 (>390)	>256 (>390)	>256 (>390)	>256 (>390)	>128 (>195)	31.24 ± 2.24
**1l**	>256 (>382)	>256 (>382)	>256 (>382)	>256 (>382)	>256 (>382)	>256 (>382)	>256 (>382)	>256 (>382)	>128 (>191)	29.75 ± 0.14
**1m**	128 (245)	32 (61.5)	64 (122)	64 (122)	32 (61.5)	32 (61.5)	64 (122)	**16** **(30.7)**	>128 (>245)	4.90 ± 0.19
**1n**	128 (239)	64 (119)	128 (239)	256 (478)	**16** **(29.9)**	**32** **(59.9)**	64 (119)	**16** **(29.9)**	>128 (>239)	4.99 ± 0.99
**1o**	>256 (>466)	128 (233)	>256 (>466)	>256 (>466)	**16** **(29.2)**	**32** **(58.3)**	256 (466)	**8** **(14.6)**	>128 (>233)	4.71 ± 0.07
**1p**	>256 (>455)	256 (455)	>256 (>455)	>256 (>455)	**8** **(14.2)**	**64** **(113)**	256 (455)	**16** **(28.4)**	>128 (>227)	6.32 ± 0.58
**AMP**	2 (5.72)	16 (45.8)	>16 (>45.8)	>16 (>45.8)	4 (11.5)	4 (11.5)	4 (11.5)	2 (5.72)	–	>30
**CPX**	0.5 (1.51)	>16 (>45.8)	>16 (>45.8)	>16 (>45.8)	1 (3.02)	1 (3.02)	1 (3.02)	64 (193)	–	0.71 ± 0.09
**VAN**	1 (0.69)	2 (1.38)	1 (0.69)	1 (0.69)	–	512 (353)	512 (353)	1024 (706)	–	>30
**5-FC**	–	–	–	–	–	–	–	–	1 (7.75)	–
**CMP**	–	–	–	–	–	–	–	–	–	0.20 ± 0.07
**SDS**	128 (443)	128 (443)	64 (222)	64 (222)	128 (443)	256 (887)	128 (443)	128 (443)	–	148.43 ± 8.42
**CRB**	0.25 (0.59)	0.25 (0.59)	0.5 (1.18)	2 (4.73)	0.5 (1.18)	0.5 (1.18)	0.25 (0.59)	0.5 (1.18)	–	1.73 ± 0.08
**CTC**	0.25 (0.63)	0.25 (0.63)	0.5 (1.26)	1 (2.53)	1 (2.53)	0.5 (1.26)	0.5 (1.26)	0.5 (1.26)	–	1.29 ± 0.07
**PRC**	>256 (>1083)	>256 (>1083)	>256 (>1083)	>256 (>1083)	>256 (>1083)	>256 (>1083)	>256 (>1083)	>256 (>1083)	–	>300
**TRC**	>256 (>1030)	>256 (>1030)	>256 (>1030)	>256 (>1030)	>256 (>1030)	>256 (>1030)	>256 (>1030)	>256 (>1030)	–	>300
**TEC**	>256 (>968)	>256 (>968)	>256 (>968)	>256 (>968)	>256 (>968)	>256 (>968)	>256 (>968)	>256 (>968)	–	224.13 ± 6.52

SA *= S. aureus* ATCC 29213; MRSA1–3 *=* clinical isolates of methicillin-resistant *S. aureus* 63718, SA 630, SA 3202 (National Institute of Public Health, Prague, Czech Republic); EF = *E. faecalis* ATCC 29213; VRE1–3 = vancomycin-resistant enterococci VRE 342B, VRE 368, VRE 725B [56]; CA = *C albicans* CCM 8261; AMP = ampicillin, CPX = ciprofloxacin, VAN = vancomycin, 5-FC = flucytosine, CMP = camptothecin, SDS = sodium dodecyl sulfate, CRB = carbethopendecinium bromide, CTC = cetalkonium chloride, PRC = procaine, TRC = trimecaine, TEC = tetracaine.

**Table 3:** Effect of tested compounds in combination with clinically used antibiotics. The MICs (μg/mL) of each antibacterial drug alone and observed in the synergy experiment are shown in parentheses. In the case of additivity and synergy, the concentrations of the tested compound/clinically used antibiotic (µg/mL) providing this effect are shown.

Isolate	Combination (MIC (μg/mL))	FIC Index	Comb. Effect (MICs (μg/mL))
MRSA 63718	Comp. **1g** + CPX (16/16)	1.000	IND
	Comp. **1h** + CPX (8/16)	0.531	ADD 0.25/0.032
	Comp. **1g** + OXA (8/512)	0.500	**SYN 2/128**
	Comp. **1h** +OXA (4/512)	0.562	ADD 2/32; 1/256
MRSA SA 3202	Comp. **1g** + CPX (16/16)	1.000	IND
	Comp. **1h** + CPX (8/16)	1.000	IND
	Comp. **1g** + OXA (8/512)	1.000	IND
	Comp. **1h**+ OXA (8/512)	0.750	ADD 4/128
VRE 342B	Comp. **1g** + VAN (8/1024)	0.625	ADD 4/128
	Comp. **1h** + VAN (8/1024)	0.500	**SYN 2/256**
	Comp. **1g** + GEN (8/32)	0.750	ADD 4/8
	Comp. **1h**+ GEN (8/32)	1.000	IND
VRE 368	Comp. **1g** + VAN (8/512)	0.750	ADD 4/128
	Comp. **1h** + VAN (4/512)	0.750	ADD 2/256
	Comp. **1g** + GEN (8/64)	1.000	IND
	Comp. **1h**+ GEN (2/128)	1.016	IND

FIC = fractional inhibitory concentration; IND = indifference; ADD = additivity; SYN = synergy; CPX = ciprofloxacin; OXA = oxacillin; VAN = vancomycin; GEN = gentamicin.

**Table 4:** Change in viable counts (log10 colony-forming unit (CFU)/mL) of staphylococci and enterococci strains following incubation for 24 h with compound **1g**. Bactericidal effect is expressed in bold.

Strain	Conc.	Log10 Difference in CFU/mL from Inoculum			
		4 h	6 h	8 h	24 h
**SA**	1 × MIC	1.54	1.98	2.33	2.74
	2 × MIC	0.87	0.98	1.26	2.24
	4 × MIC	−0.26	−0.04	0.10	1.85
**MRSA1**	1 × MIC	0.00	0.66	1.00	1.99
	2 × MIC	−1.33	−0.93	−0.26	1.29
	4 × MIC	−2.57 *	−1.99 *	−1.26 *	1.21
**EF**	1 × MIC	−1.63	−1.01	−0.61	0.27
	2 × MIC	−3.37 *	−3.43 *	−2.68	1.04
	4 × MIC	−5.62 *	−5.14 *	−5.92 *	−0.31
**VRE1**	1 × MIC	−0.14	0.97	1.01	1.11
	2 × MIC	−0.36	−1.89	−0.64	1.51
	4 × MIC	−0.92	−1.00	−1.09	0.77
**VRE2**	1 × MIC	−2.00	0.29	0.25	−0.16
	2 × MIC	−2.34	−1.97	−1.01	0.32
	4 × MIC	−2.72 *	−2.71 *	−2.45	0.97
**VRE3**	1 × MIC	−0.09	−0.29	0.19	2.41
	2 × MIC	−0.75	−0.74	0.56	0.43
	4 × MIC	−1.85	−1.85	−2.15	−2.27

SA *= S. aureus* ATCC 29213; MRSA1 *= S. aureus* 63718, EF = *E. faecalis* ATCC 29213, VRE1 = VRE 342B; VRE2 = VRE 368; VRE3 = VRE 725B. * Statistically significant decrease of CFU/mL (*p* < 0.05) compared to growth control in time 0.

**Table 5:** Change in viable counts (log10 CFU/mL) of staphylococci and enterococci strains following incubation for 24 h with compound **1h**.

Strain	Conc.	Log10 Difference in CFU/mL from Inoculum			
		4 h	6 h	8 h	24 h
**SA**	1 × MIC	0.08	0.31	0.50	2.07
	2 × MIC	0.05	0.10	0.05	2.01
	4 × MIC	−0.25	−0.13	−0.08	1.93
**MRSA1**	1 × MIC	−0.73 *	−0.11	0.98	1.58
	2 × MIC	−0.92 *	−0.42 *	−0.13	1.62
	4 × MIC	−2.48 *	−2.40 *	−2.08 *	1.35
**EF**	1 × MIC	−0.64	0.16	0.24	0.34
	2 × MIC	−1.87	−1.36	−0.86	0.42
	4 × MIC	−2.57 *	−2.23 *	−1.62	0.49
**VRE1**	1 × MIC	0.07	1.08	−0.08	1.42
	2 × MIC	0.09	0.41	−0.02	0.47
	4 × MIC	−0.78	−0.99	−0.66	0.85
**VRE2**	1 × MIC	0.46	0.71	1.25	1.13
	2 × MIC	0.28	0.54	−0.37	0.66
	4 × MIC	−0.32	0.05	0.22	0.37
**VRE3**	1 × MIC	−0.70	0.08	−0.05	0.92
	2 × MIC	−1.81	−1.31	−0.81	0.95
	4 × MIC	−1.75 *	−0.76	−0.76	1.08

SA *= S. aureus* ATCC 29213; MRSA1 *= S. aureus* 63718, EF = *E. faecalis* ATCC 29213, VRE1 = VRE 342B; VRE2 = VRE 368; VRE3 = VRE 725B. * Statistically significant decrease of CFU/mL *(p* < 0.05) compared to growth control in time 0.

**Table 6:** Change in viable counts (log10 CFU/mL) of staphylococci strains following incubation for 24 h with vancomycin.

Strain	Conc.	Log10 Difference in CFU/mL from Inoculum			
		4 h	6 h	8 h	24 h
**SA**	1 × MIC	−0.37	−0.65	−0.84	2.90
	2 × MIC	−0.26	−0.59	−1.12	−3.67
	4 × MIC	−0.27	−0.38	−1.18	−5.47
**MRSA1**	1 × MIC	−1.00	−1.50	−1.76	0.55
	2 × MIC	−0.91	−1.43	−1.97	−5.18
	4 × MIC	−1.31	−1.75	−3.89	−5.19

SA = *S. aureus* ATCC 29213; MRSA1 = *S. aureus* 63718.

**Table 7:** Comparison of minimum inhibitory concentrations (MICs), minimum biofilm inhibitory concentrations (MBIC_80_), and minimum biofilm eradication concentrations (MBEC_80_) (µg/mL) of tested compounds and standards.

Sample	(µg/mL)		
	MIC	MBIC_80_	MBEC_80_
**1f**	16	32	64
**1g**	8	8	16
**1h**	8	8	16
**SDS**	128	32	128
**CRB**	0.25	0.25	64
**CTC**	0.25	0.25	>256
**PRC**	>256	>256	>256
**TRC**	>256	>256	>256
**TEC**	>256	>256	>256

SDS = sodium dodecyl sulfate; CRB = carbethopendecinium bromide; CTC = cetalkonium chloride; PRC = procaine; TRC = trimecaine; TEC = tetracaine.

## Supplementary Materials

The following are available online at https://www.mdpi.com/2079-6382/9/2/64/s1, Figure S1: Dynamics of antibacterial activity of compound **1g** against staphylococci (A,B) and enterococci (C–F), Figure S2: Dynamics of antibacterial activity of compound **1h** against staphylococci (A,B) and enterococci (C–F).

## References

[1] Dastidar S.G., Kristiansen J.E., Molnar J., Amaral L. (2013). Role of phenothiazines and structurally similar compounds of plant origin in the fight against infections by drug resistant bacteria. Antibiotics.

[2] Hamad M., Al-Marzooq F., Orive G., Al-Tel T.H. (2019). Superbugs but no drugs: Steps in averting a post-antibiotic era. Drug Discov. Today.

[3] Kristiansen J.E., Amaral L. (1997). The potential management of resistant infections with non-antibiotics. J. Antimicrob. Chemoth..

[4] Amaral L., Lorian V. (1991). Effects of chlorpromazine on the cell envelope proteins of Escherichia coli. Antimicrob. Agents Chemother..

[5] Chattopadhyay D., Das S.K., Patra A.R., Bhattacharya S.K., Ahmad I., Aqil F. (2009). Non-Antibiotics—An alternative for microbial resistance: Scope and hope. New Strategies Combating Bacterial Infection.

[6] Schmidt R.M., Rosenkranz H.S. (1970). Antimicrobial activity of local anaesthetics: Lidocaine and procaine. J. Infect. Dis..

[7] de Silva S.S., Carvalho J.W.P., Aires C.P., Nitschke M. (2017). Disruption of Staphylococcus aureus biofilms using rhamnolipid biosurfactants. J. Dairy Sci..

[8] Sriram M.I., Kalishwaralal K., Deepak V., Gracerosepat R., Srisakthi K., Gurunathan S. (2011). Biofilm inhibition and antimicrobial action of lipopeptide biosurfactant produced by heavy metal tolerant strain Bacillus cereus NK1. Colloids Surf. B. Biointerfaces.

[9] Meylheuc T., Van Oss C.J., Bellon-Fontaine M.N. (2001). Adsorption of biosurfactant on solid surfaces and consequences regarding the bioadhesion of Listeria monocytogenes LO28. J. Appl. Microbiol..

[10] Wieczorek D., Dobrowolski A., Staszak K., Kwasniewska D., Dubyk P. (2016). Surface and antimicrobial activity of sulfobetaines. J. Surfactants Deterg..

[11] Rewak-Soroczynska J., Paluch E., Siebert A., Szalkiewicz K., Oblak E. (2019). Biological activity of glycine and alanine derivatives of quaternary ammonium salts (QASs) against micro-organisms. Lett. Appl. Microbiol..

[12] Aydin O.N., Eyigor M., Aydin N. (2001). Antimicrobial activity of ropivacaine and other local anaesthetics. Eur. J. Anaesthesiol..

[13] Mullin G.S., Rubinfeld R.S. (1997). The antibacterial activity of topical anesthetics. Cornea.

[14] Kesici S., Demirci M., Kesici U. (2019). Bacterial inhibition efficiency of prilocaine and bupivacaine. Int. Wound J..

[15] Pina-Vaz C., Rodrigues A.G., Sansonetty F., Martinez-De-Oliveira J., Fonseca A.F., Mardh P.A. (2000). Antifungal activity of local anesthetics against Candida species. Infect. Dis. Obstet. Gynecol..

[16] Srisatjaluk R.L., Klongnoi B., Wongsirichat N. (2016). Antimicrobial effect of topical local anesthetic spray on oral microflora. J. Dent. Anesth. Pain. Med..

[17] Johnson S.M., Saint J., Barbara E., Dine A.P. (2008). Local anesthetics as antimicrobial agents: A review. Surg. Infect..

[18] Mutlu E. (2018). In vitro investigation of the antibacterial effects of lidocaine and bupivacaine alone and in combinations with fentanyl. Turk. Klin. J. Med. Sci..

[19] Kramer A., Sorgatz K., Hoppe H., Meyer F.U. (1994). Bacteriostatic and antiseptic efficacy of selected anaesthetics individually and in combination with an antiseptic mouthwash. Hygiene Medizin.

[20] Razavi B.M., Fazly-Bazzaz B.S. (2019). A review and new insights to antimicrobial action of local anesthetics. Eur. J. Clin. Microbiol. Infect. Dis..

[21] Abdelli F., Jardak M., Elloumi J., Stien D., Cherif S., Mnif S., Aifa S. (2019). Antibacterial, anti-adherent and cytotoxic activities of surfactin (s) from a lipolytic strain Bacillus safensis F4. Biodegradation.

[22] Lewis K. (2001). Riddle of biofilm resistance. Antimicrob. Agents Chemother..

[23] Jamal M., Ahmad W., Andleeb S., Jalil F., Imran M., Nawaz M.A., Kamil M.A. (2018). Bacterial biofilm and associated infections. J. Chin. Med. Assoc..

[24] Scott VanEpps J., Younger J.G. (2016). Implantable device related infection. Shock.

[25] Azevedo M.M., Cobrado L., Silva-Dias A., Pina-Vaz C., Rodrigues A.G., Atta-ur-Rahman (2017). Prevention and eradication of biofilm in medical indwelling devices. Frontiers in Clinical Drug Research—Anti Infectives.

[26] Khatoon Z., McTiernan C.D., Suuronen E.J., Mah T.F., Alarcona E.I. (2018). Bacterial biofilm formation on implantable devices and approaches to its treatment and prevention. Heliyon.

[27] Mandakhalikar K.D., Chua R.R., Tambyah P.A. (2016). New technologies for prevention of catheter associated urinary tract infection. Curr. Treat. Options Infect. Dis..

[28] Gominet M., Compain F., Beloin C., Lebeaux D. (2017). Central venous catheters and biofilms: Where do we stand in 2017?. APMIS.

[29] Menezes F.G., Correa L., Medina-Pestana J.O., Aguiar W.F., Camargo L.F.A. (2019). A randomized clinical trial comparing Nitrofurazone-coated and uncoated urinary catheters in kidney transplant recipients: Results from a pilot study. Transpl. Infect. Dis..

[30] Doherty W. (1999). Instillagel: An anaesthetic antiseptic gel for use in catheterization. Br. J. Nurs..

[31] Wilson M.C.R. (2009). Biofilm and other causes of pain in catheterization. Br. J. Community Nurs..

[32] Lai N.M., Chaiyakunapruk N., Lai N.A., O’Riordan E., Pau W.S.C., Saint S. (2016). Catheter impregnation, coating or bonding for reducing central venous catheter-related infections in adults. Cochrane Database Syst. Rev..

[33] Chong H.Y., Lai N.M., Apisarnthanarak A., Chaiyakunapruk N. (2017). Comparative efficacy of antimicrobial central venous catheters in reducing catheter-related bloodstream infections in adults: Abridged cochrane systematic review and network meta-analysis. Clin. Infect. Dis..

[34] Wang H., Tong H., Liu H., Wang Y., Wang R., Gao H., Yu P., Lv Y., Chen S., Wang G. (2018). Effectiveness of antimicrobial-coated central venous catheters for preventing catheter-related blood-stream infections with the implementation of bundles: A systematic review and network meta-analysis. Ann. Intensive Care.

[35] Dang F.P., Li H.J., Tian J.H. (2019). Comparative efficacy of 13 antimicrobial dressings and different securement devices in reducing catheter-related bloodstream infections: A Bayesian network meta-analysis. Medicine.

[36] Yeung S.S.T., Loshak H. (2019). Coated and Uncoated Central Venous Catheters: A Review of Comparative Clinical Effectiveness and Safety.

[37] Majeed A., Sagar F., Latif A., Hassan H., Iftikhar A., Darouiche R.O., Mohajer M.A. (2019). Does antimicrobial coating and impregnation of urinary catheters prevent catheter-associated urinary tract infection? A review of clinical and preclinical studies. Expert Rev. Med. Devices..

[38] Al-Qahtani M., Safan A., Jassim G., Abadla S. (2019). Efficacy of anti-microbial catheters in preventing catheter associated urinary tract infections in hospitalized patients: A review on recent updates. J. Infect. Public Health.

[39] Monteiro C., Costa F., Pirttila A.M., Tejesvi M.V., Cristina M., Martins L. (2019). Prevention of urinary catheter-associated infections by coating antimicrobial peptides from crowberry endophytes. Sci. Rep..

[40] Andersen M.J., Flores-Mireles A.L. (2020). Urinary catheter coating modifications: The race against catheter-associated infections. Coatings.

[41] Otto M. (2008). Staphylococcal biofilms. Curr. Top. Microbiol. Immunol..

[42] Malheiro J., Simoes M., Deng Y., Lv W. (2017). Antimicrobial resistance of biofilms in medical devices. Biofilms and Implantable Medical Devices.

[43] Larsen T., Fiehn N.E. (1996). Resistance of Streptococcus sanguis biofilms to antimicrobial agents. APMIS.

[44] Olsen I. (2015). Biofilm-specific antibiotic tolerance and resistance. Eur. J. Clin. Microbiol. Infect. Dis..

[45] Pace J.L., Rupp M.E., Finch R.G. (2006). Biofilms, Infection, and Antimicrobial Therapy.

[46] Vetas D., Dimitropoulou E., Mitropoulou G., Kourkoutas Y., Giaouris E. (2017). Disinfection efficiencies of sage and spearmint essential oils against planktonic and biofilm Staphylococcus aureus cells in comparison with sodium hypochlorite. Int. J. Food Microbiol..

[47] Kwiecinska-Pirog J., Bogiel T., Gospodarek E. (2013). Effects of ceftazidime and ciprofloxacin on biofilm formation in Proteus mirabilis rods. J. Antibiot..

[48] Jampilek J. (2018). Design and discovery of new antibacterial agents: Advances, perspectives, challenges. Curr. Med. Chem..

[49] Singha P., Locklin J., Handa H. (2017). A review of the recent advances in antimicrobial coatings for urinary catheters. Acta Biomater..

[50] Walters M.C., Roe F., Bugnicourt A., Franklin M.J., Stewart P.S. (2003). Contributions of antibiotic penetration, oxygen limitation, and low metabolic activity to tolerance of Pseudomonas aeruginosa biofilms to ciprofloxacin and tobramycin. Antimicrob. Agents Chemother..

[51] Hall C.W., Mah T.F. (2017). Molecular mechanisms of biofilm-based antibiotic resistance and tolerance in pathogenic bacteria. FEMS Microbiol. Rev..

[52] Borrielo G., Richards L., Ehrlich G.D., Stewart P.S. (2006). Arginine or nitrate enhances antibiotic susceptibility of Pseudomonas aeruginosa in biofilms. Antimicrob. Agents Chemother..

[53] Van Ecker H., Sass A., Bazzini S., De Roy K., Udine C., Messiaen T., Coenye T. (2013). Biofilm-grown Burkholderia cepacia complex cells survive antibiotic treatment by avoiding production of reactive oxygen species. PLoS ONE.

[54] Malik I., Csollei J., Solovic I., Pospisilova S., Michnova H., Jampilek J., Cizek A., Kapustikova I., Curillova J., Pechacova M. (2018). Dibasic derivatives of phenylcarbamic acid against mycobacterial strains: Old drugs and new tricks?. Molecules.

[55] Csollei J., Buciova L., Cizmarik J., Kopacova L. (1993). Studies of local anaesthetics CXII. Preparation and activity of dibasic alkylesters of 2-, and 3-alkoxy-substituted phenylcarbamic acids. Ceskoslov. Farm.

[56] Oravcova V., Zurek L., Townsend A., Clark A.B., Ellis J.C., Cizek A. (2014). American crows as carriers of vancomycin-resistant enterococci with vanA gene. Environ. Microbiol..

[57] Zadrazilova I., Pospisilova S., Masarikova M., Imramovsky A., Monreal-Ferriz J., Vinsova J., Cizek A., Jampilek J. (2015). Salicylanilide carbamates: Promising antibacterial agents with high in vitro activity against methicillin-resistant Staphylococcus aureus (MRSA). Eur. J. Pharm. Sci..

[58] Tengler J., Kapustikova I., Pesko M., Govender R., Keltosova S., Mokry P., Kollar P., O´Mahony J., Coffey A., Kralova K. (2013). Synthesis and biological evaluation of 2-hydroxy-3-[(2-aryloxyethyl)amino]propyl 4-[(alkoxycarbonyl)amino]benzoates. Sci. World J..

[59] Imramovsky A., Pesko M., Kralova K., Vejsova M., Stolarikova J., Vinsova J., Jampilek J. (2011). Investigating spectrum of biological activity of 4- and 5-chloro-2-hydroxy-N-[2-(arylamino)-1-alkyl-2-oxoethyl]- benzamides. Molecules.

[60] Pauk K., Zadrazilova I., Imramovsky A., Vinsova J., Pokorna M., Masarikova M., Cizek A., Jampilek J. (2013). New derivatives of salicylamides: Preparation and antimicrobial activity against various bacterial species. Bioorg. Med. Chem..

[61] Pere P., Lindgren L., Vaara M. (1999). Poor antibacterial effect of ropivacaine: Comparison with bupivacaine. Anesthesiology.

[62] ROCHE (2011). Cell Proliferation Reagent WST-1. https://www.sigmaaldrich.com/content/dam/sigma-aldrich/docs/Roche/Bulletin/1/cellprorobul.pdf.

[63] Kos J., Nevin E., Soral M., Kushkevych I., Gonec T., Bobal P., Kollar P., Coffey A., O´Mahony J., Liptaj T. (2015). Synthesis and antimycobacterial properties of ring-substituted 6-hydroxynaphthalene- 2-carboxanilides. Bioorg. Med. Chem..

[64] Lim E.J., Yoon Y.J., Heo J., Lee T.H., Kim Y.H. (2018). Ciprofloxacin enhances TRAIL-induced apoptosis in lung cancer cells by upregulating the expression and protein stability of death receptors through CHOP expression. Int. J. Mol. Sci..

[65] Suffness M., Douros J. (1982). Current status of the NCI plant and animal product program. J. Nat. Prod..

[66] Abdi H., Williams L.J. (2010). Principal component analysis. WIREs Comp. Stat..

[67] van den Berg R.A., Hoefsloot H.C., Westerhuis J.A., Smilde A.K., van der Werf M.J. (2006). Centering, scaling, and transformations: Improving the biological information content of metabolomics data. BMC Genom..

[68] Bro R., Smilde A.K. (2014). Principal component analysis. Anal. Methods.

[69] Eriksson L., Jaworska J., Worth A.P., Cronin M.T., McDowell R.M., Gramatica P. (2003). Methods for reliability and uncertainty assessment and for applicability evaluations of classification- and regression-based QSARs. Environ. Health Perspect..

[70] Jackson D.A. (1993). Stopping rules in principal components analysis: A comparison of heuristical and statistical approaches. Ecology.

[71] Hauke J., Kossowski T. (2011). Comparison of values of Pearson’s and Spearman’s correlation coefficients on the same sets of data. Quaest. Geograph..

[72] Schwalbe R., Steele-Moore L., Goodwin A.C. (2007). Antimicrobial Susceptibility Testing Protocols.

[73] Bonapace C.R., Bosso J.A., Friedrich L.V., White R.L. (2002). Comparison of methods of interpretation of checkerboard synergy testing. Diagn. Microbiol. Infect. Dis..

[74] Breukink E., De Kruijff B. (2006). Lipid II as a target for antibiotics. Nat. Rev. Drug Discov..

[75] Tipper D.J., Strominger J.L. (1965). Mechanism of action of penicillins: A proposal based on their structural similarity to acyl-D-alanyl-D-alanine. Proc. Natl. Acad. Sci. USA.

[76] Arias C.A., Murray B.E. (2012). The rise of the Enterococcus: Beyond vancomycin resistance. Nat. Rev. Microbiol..

[77] Stapleton P.D., Taylor P.W. (2002). Methicillin resistance in Staphylococcus aureus: Mechanisms and modulation. Sci. Prog..

[78] Cetinkaya Y., Falk P., Mayhall C.G. (2000). Vancomycin-resistant enterococci. Clin. Microbiol. Rev..

[79] Cha J.O., Park Y.K., Lee Y.S., Chung G.T. (2011). In vitro biofilm formation and bactericidal activities of methicillin-resistant Staphylococcus aureus clones prevalent in Korea. Diagn. Microbiol. Infect. Dis..

[80] Devi K.P., Nisha S.A., Sakthivel R., Pandian S.K. (2010). Eugenol (an essential oil of clove) acts as an antibacterial agent against Salmonella typhi by disrupting the cellular membrane. J. Ethnopharmacol..

[81] Pospisilova S., Kos J., Michnova H., Kapustikova I., Strharsky T., Oravec M., Moricz A.M., Bakonyi J., Kauerova T., Kollar P. (2018). Synthesis and spectrum of biological activities of novel N-arylcinnamamides. Int. J. Mol. Sci..

[82] Clinical and Laboratory Standards Institute (2012). Performance Standards for Antimicrobial Susceptibility Testing.

[83] Ringner M. (2008). What is principal component analysis?. Nat. Biotechnol..

