# Supplementary material for: Dibasic Derivatives of Phenylcarbamic Acid as Prospective Antibacterial Agents Interacting with Cytoplasmic Membrane
Source: Antibiotics (Basel). 2020 Feb 6;9(2):64. doi: 10.3390/antibiotics9020064 (PMC7168207; doi:10.3390/antibiotics9020064)
Supplement: Supplementary file 1 [file antibiotics-09-00064-s001.pdf]

Supplementary Materials

Dibasic Derivatives of Phenylcarbamic Acid as Prospective Antibacterial Agents Interacting with Cytoplasmic Membrane

Sarka Pospisilova [1], Ivan Malik [2,*], Kristyna Bezouskova [3], Tereza Kauerova [4], Peter Kollar [4], Jozef Csollei [5], Michal Oravec [6], Alois Cizek [3] and Josef Jampilek [1,7,*]

1 Regional Centre of Advanced Technologies and Materials, Faculty of Science, Palacky University, Slechtitelu 27, 783 71 Olomouc, Czech Republic, sharka.pospisilova@gmail.com (S.P.)
2 Department of Pharmaceutical Chemistry, Faculty of Pharmacy, Comenius University in Bratislava, Odbojarov 10, 832 32 Bratislava, Slovak Republic
3 Department of Infectious Diseases and Microbiology, Faculty of Veterinary Medicine, University of Veterinary and Pharmaceutical Sciences, Palackeho 1946/1, 612 42 Brno, Czech Republic; bezouskova.kristyna@gmail.com (K.B.); cizeka@vfu.cz (A.C.)
4 Department of Human Pharmacology and Toxicology, Faculty of Pharmacy, University of Veterinary and Pharmaceutical Sciences, Palackeho 1, 61242 Brno, Czech Republic; tereza.kauerova@gmail.com (T.K.); kollarp@vfu.cz (P.K.)
5 Department of Chemical Drugs, Faculty of Pharmacy, University of Veterinary and Pharmaceutical Sciences in Brno, Palackeho 1946/1, 612 42 Brno, Czech Republic; csolleij@vfu.cz
6 Global Change Research Institute CAS, Belidla 986/4a, 603 00 Brno, Czech Republic; oravec.m@czechglobe.cz
7 Institute of Neuroimmunology, Slovak Academy of Sciences, Dubravska cesta 9, 845 10 Bratislava, Slovak Republic
* Correspondence: malikivan001@gmail.com (I.M.); josef.jampilek@gmail.com (J.J.)

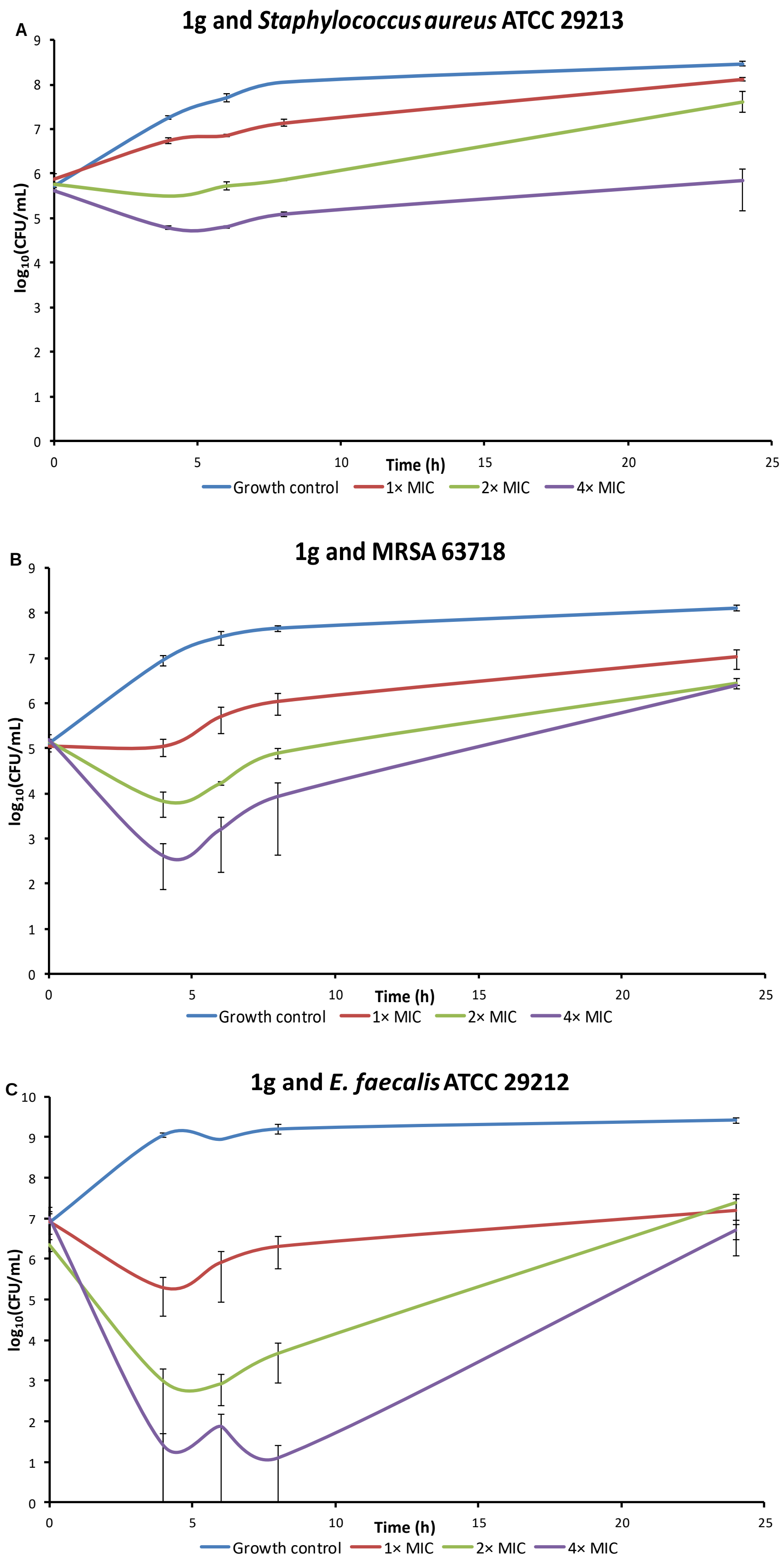
A
1g and *Staphylococcus aureus* ATCC 29213
log10(CFU/mL)
Time (h)
Growth control
1× MIC
2× MIC
4× MIC
B
1g and MRSA 63718
log10(CFU/mL)
Time (h)
Growth control
1× MIC
2× MIC
4× MIC
C
1g and *E. faecalis* ATCC 29212
log10(CFU/mL)
Time (h)
Growth control
1× MIC
2× MIC
4× MIC

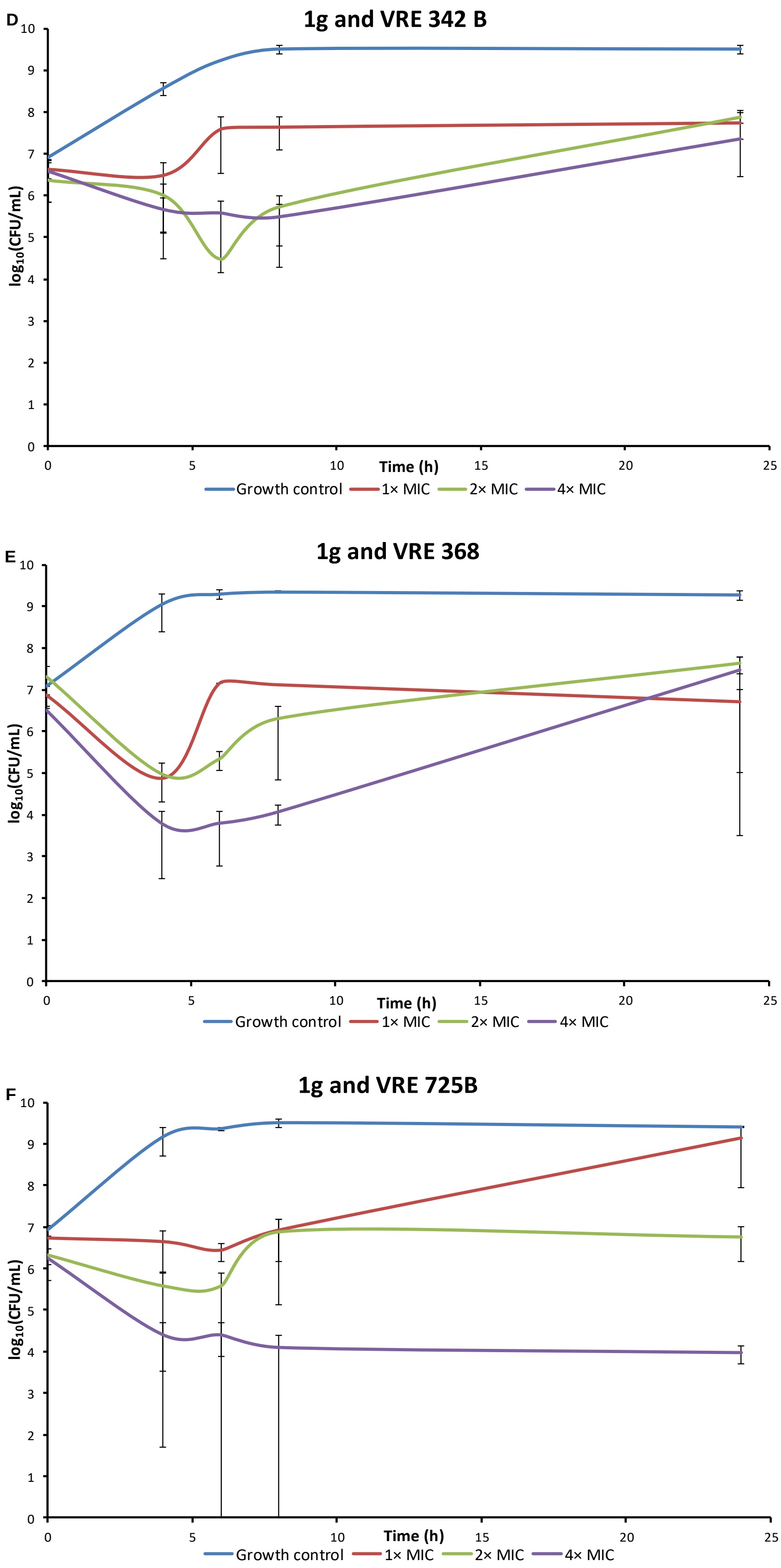

Figure S1. Dynamics of antibacterial activity of 1-[3-(dipropylammonio)-2-({[3-(hexyloxy)phenyl] carbamoyl}oxy)propyl]pyrrolidinium dichloride (**1g**) against staphylococci (**A**, **B**) and enterococci (**C**–**F**).

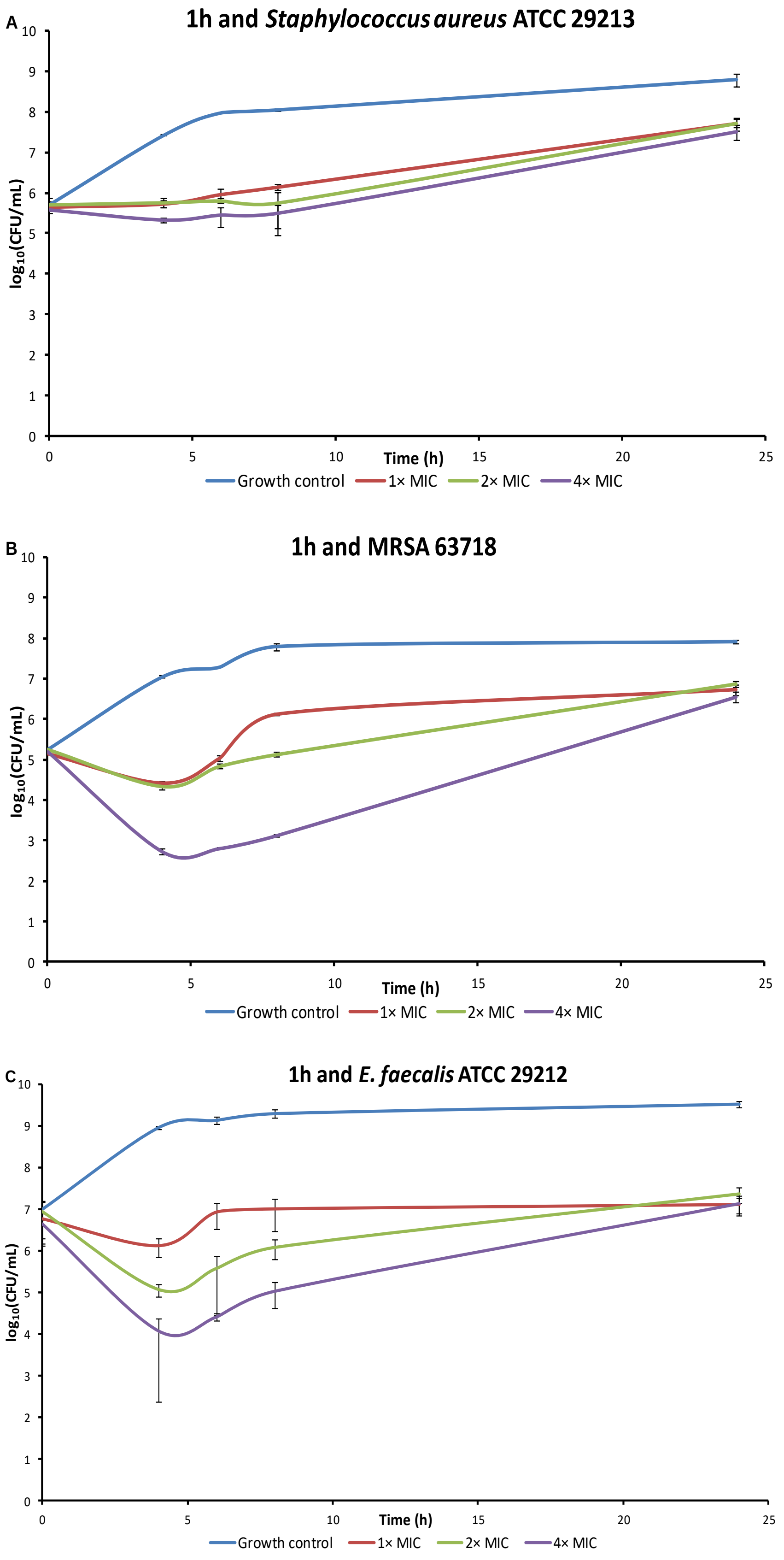
A
1h and *Staphylococcus aureus* ATCC 29213
log10(CFU/mL)
Time (h)
0 5 10 15 20 25
Growth control
1× MIC
2× MIC
4× MIC
B
1h and MRSA 63718
log10(CFU/mL)
Time (h)
Growth control
1× MIC
2× MIC
4× MIC
C
1h and *E. faecalis* ATCC 29212
log10(CFU/mL)
Time (h)
Growth control
1× MIC
2× MIC
4× MIC

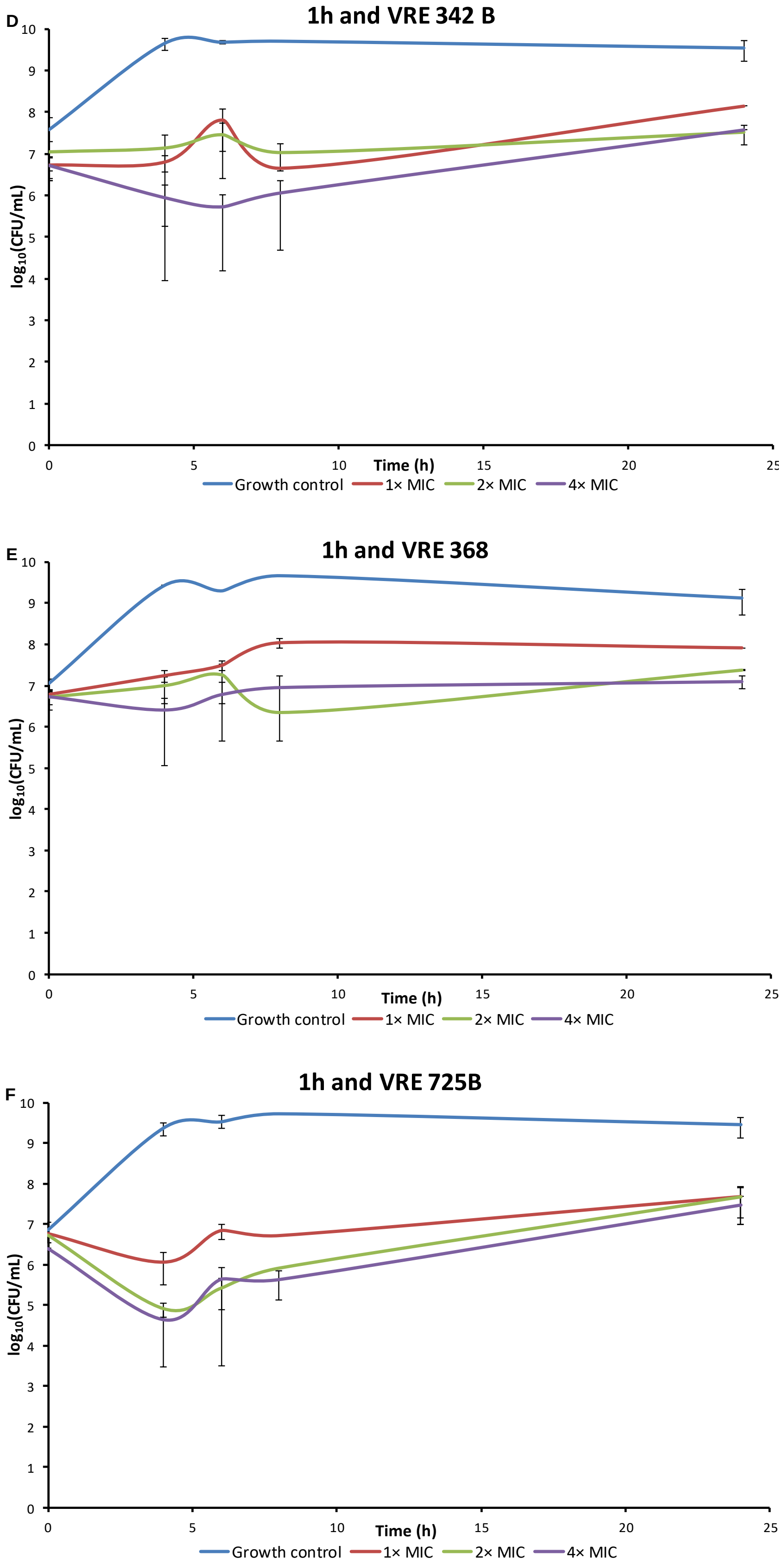

Figure S2. Dynamics of antibacterial activity of 1-[3-(dipropylammonio)-2-({[3-(heptyloxy)phenyl]-carbamoyl}oxy)- propyl]pyrrolidinium dichloride (**1h**) against staphylococci (**A**, **B**) and enterococci (**C**–**F**).
